# Molecular Cytology of ‘Little Animals’: Personal Recollections of *Escherichia coli* (and *Bacillus subtilis*)

**DOI:** 10.3390/life13081782

**Published:** 2023-08-21

**Authors:** Nanne Nanninga

**Affiliations:** Molecular Cytology, Swammerdam Institute for Life Sciences (SILS), University of Amsterdam, 1098 XH Amsterdam, The Netherlands; nannenanninga@gmail.com

**Keywords:** electron microscopy, confocal microscopy, image processing, bacteria, divisome, elongasome, PIPS

## Abstract

This article relates personal recollections and starts with the origin of electron microscopy in the sixties of the previous century at the University of Amsterdam. Novel fixation and embedding techniques marked the discovery of the internal bacterial structures not visible by light microscopy. A special status became reserved for the freeze-fracture technique. By freeze-fracturing chemically fixed cells, it proved possible to examine the morphological effects of fixation. From there on, the focus switched from bacterial structure as such to their cell cycle. This invoked bacterial physiology and steady-state growth combined with electron microscopy. Electron-microscopic autoradiography with pulses of [^3^H] Dap revealed that segregation of replicating DNA cannot proceed according to a model of zonal growth (with envelope-attached DNA). This stimulated us to further investigate the sacculus, the peptidoglycan macromolecule. In particular, we focused on the involvement of penicillin-binding proteins such as PBP2 and PBP3, and their role in division. Adding aztreonam (an inhibitor of PBP3) blocked ongoing divisions but not the initiation of new ones. A PBP3-independent peptidoglycan synthesis (PIPS) appeared to precede a PBP3-dependent step. The possible chemical nature of PIPS is discussed.

## 1. Introduction

**Figure 1:**
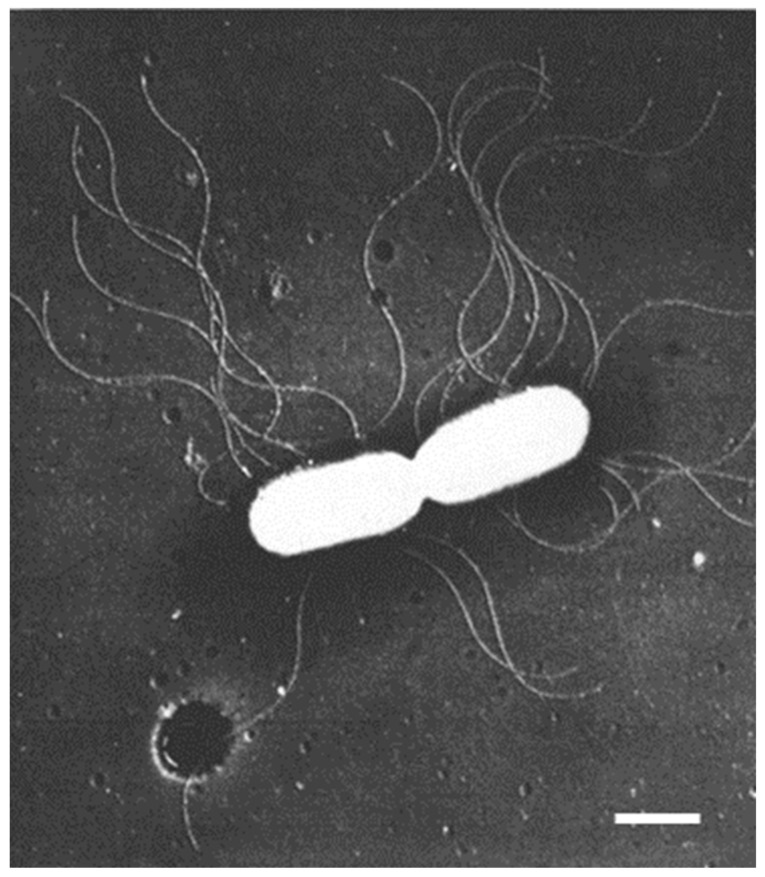
*Bacterium herbicola* with peritrichous flagella after shadowcasting. Bar, 1 µm. Source: Figure 3 from [1]. Copyright © Elsevier Permissions Helpdesk.

In the early sixties of the previous century, I was involved in photosynthetic experiments with chloroplasts isolated from succulents. The results deviated from those obtained with spinach chloroplasts (the spinach was purchased from a nearby greengrocer). One of the explanations which came to my mind was the possibility that the structure of succulent chloroplasts might differ from that of the standard spinach chloroplasts. With this idea, I paid a visit to the Laboratory of Electron Microscopy of the University of Amsterdam. This laboratory was headed by Dr. Woutera van Iterson, who came from the Technical University of Delft, where she pioneered very successfully the use of the electron microscope for the study of bacterial flagella (for instance, *Bacterium herbicola* in Houwink and van Iterson, 1950 [1]; Figure 1). She continued her work on bacterial ultrastructure in Amsterdam.

I do not remember our conversation, but presumably I could not explain to her what kind of difference to expect in chloroplast structure, neither how this might explain my results with chloroplasts of succulent plants. Anyway, she more or less convinced me that it would be good to become familiar with the electron microscope and electron microscopic techniques and to begin with bacteria. And so I started with electron microscopy of wall-less L-forms of *Proteus vulgaris*. The aim was to study the organization of bacterial cytoplasm. The idea was that this would be facilitated by the removal of DNA. I found out that, after chemical fixation with osmium tetroxide, I could still remove its DNA with DNase. Later on (after 1965; see below), I felt that a descriptive approach to cytoplasmic structure should be complemented by an analysis of its components, that is, ribosomes and their subunits. So, I embarked on sucrose gradient centrifugation to isolate 50 S ribosomal subunits. I remember my excitement when I obtained my first purified preparation; however, my recollections in this field are less suitable for the current article.

In 1965, I completed my biological studies, and at the same time I obtained a position at the Laboratory of Electron Microscopy of the University of Amsterdam. I became heavily involved in fixation and embedding techniques, and I also learned more and more about possible pitfalls in microscopy. A recurrent theme was the concern about the reliability of the structures observed in the electron microscope. Two examples, which I shall indicate briefly here, will be shown later on. The first example refers to the significance of mesosomes (internal membranous structures visible in thin sections of Gram-positive bacteria) and the second refers to the variability of the shape of the nucleoid (the bacterial chromosome) in relation to the fixation technique employed. This latter problem led to a search for a microscopic technique to bridge the resolution gap between light and electron microscopy. It resulted in the construction of one of the first (if not the first) operational confocal scanning light microscope(s). This latter topic will be very briefly dealt with at the end of this article.

In 1970, I finished my Ph.D. study, entitled Dissecting a Bacterium (Promotor: Prof. Dr. D. Stegwee; Coreferent: Dr. W. van Iterson) [2]. In those times, the approach to bacterial anatomy was static. For instance, the question was: what does *Escherichia coli* look like after freeze-fracturing? Then, it was a valid question, because freeze-fracturing was a very novel technique and its application could provide new structural information (Nanninga, 1970 [3] and van Gool and Nanninga, 1971 [4]). However, it was quickly realized that a bacterial cell is a dynamic structure. Cells may elongate and they might start with division. In addition, their genetic material (DNA) has to replicate and segregate during the division cycle. What could electron microscopy contribute to these problems? Furthermore, a dynamic structure required handling of bacterial physiology (see below).

Before embarking on these questions, I will briefly make some remarks on early electron microscopy and then proceed with a general framework revealing the importance of electron microscopy for the study of bacterial DNA segregation and cell division. For instance, is the term mitosis applicable, and do bacteria posses a structure equivalent to the eukaryotic spindle apparatus? In fact, this was the framework in the beginning which guided us (partly in hindsight) towards our future research.

## 2. Early Electron Microscopy

### 2.1. From the Outside

Electron microscopic preparations require the presence of heavy elements to facilitate image formation by electrons. Initially, a frequently employed technique consisted of the evaporation of a heavy element (e.g., platinum) in a vacuum chamber onto a small specimen. Generally, the evaporated metal covered the specimen at a defined angle. In this way, no metal was deposited behind an object, revealing an electron-transparent shadow. Reversing the contrast of the recorded images in the darkroom produced a more natural appearance of the objects because of the black shadows, hence the term shadowcasting. Thus, for the first time, viruses could be seen and, in the case of bacteria, appendages such as flagella could be easily distinguished (Figure 1). These results clearly demonstrated the advantage of the higher resolution of the electron microscope as compared with the light microscope.

### 2.2. From Outside to Inside

However, what can be found in the interior of a bacterial cell? How does a bacterial cell compare to a eukaryotic cell? For instance, is there a nuclear membrane, an endoplasmic reticulum, or a mitochondrion or equivalents of them, and is chromosome segregation based on mitosis? Today, we largely know the answers to these questions, but in the middle of the twentieth century, very little was known.

**Figure 2:**
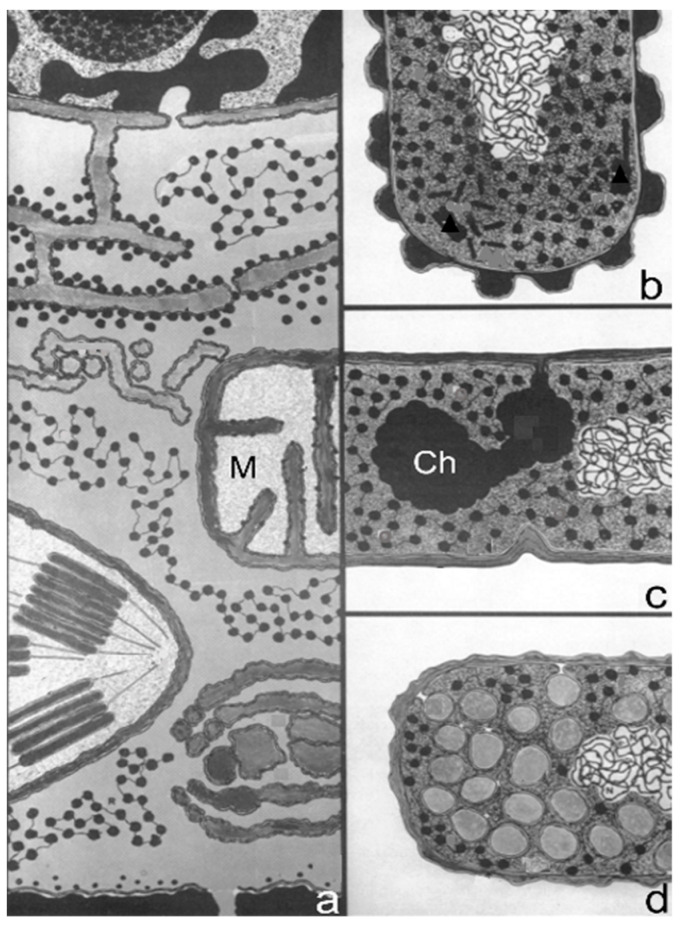
Comparison of a chloroplast-containing eukaryote (**a**) with three prokaryotes: (**b**) A Gram-negative bacterium; (**c**) A Gram-positive bacterium; and (**d**) A photosynthetic bacterium. Note the comparison of a mitochondrion M in (**a**) with a chondrioid or mesosome Ch in (**c**). Source: Modified diagram 1 from [9]. See original [9] for a more detailed description. Copyright © American Society for Microbiology.

A breakthrough was a refinement of fixation and embedding procedures to obtain reproducible images by ultrathin sections for electron microscopy (Ryter et al., 1958 [5]). Colloquially, it was denoted as R-K fixation. In addition, embedding techniques and microtomes were improved to allow very thin sections for the electron microscope. This development permitted the visualization of the bacterial cell’s interior at high resolution, thus going from outside (shadowcasting) to inside. A wealth of new information was obtained by electron microscopists studying bacterial anatomy. It is beyond the scope of this paper to provide details. However, a notable observation was the demonstration of a membranous body in the middle of the Gram-positive *Bacillus megaterium* cell and it was therefore termed the mesosome (Fitz-James, 1960 [6]). Earlier, they had been denoted as peripheral bodies by Chapman and Hillier (1953) [7]. Van Iterson (1961) [8], in a classic study, referred to them as chondrioids in the supposition that they were the bacterial equivalents of eukaryotic mitochondria (van Iterson, 1961 [8]; van Iterson, 1965 [9]; Figure 2).

### 2.3. Freeze Fracturing

A completely different approach was a method without chemical fixation: freeze-fracturing. The freeze-fracturing technique was invented by Hans Moor and coworkers from the Swiss Federal Institute of Technology, Zürich, Switzerland (Moore et al., 1961 [10]). Briefly, living specimens are frozen and thereafter fractured in vacuum. The resulting fractures could be replicated by shadowcasting and, after cleaning, the replicas could be studied in the electron microscope. It revealed insights in cellular structure by images which had never been seen before. The technique was further elaborated by a study on the “Fine structure of frozen-etched yeast cells” (Moor and Mühlethaler, 1963 [11]). An interesting observation was the finding that frozen yeast cells could continue their growth after thawing, implying that replicas were made of living cells. The interpretation appeared complicated, however, notably concerning the fracture faces of frozen membranes.

Originally, Moor and Mühlethaler (1963) [11] entertained the idea that a fractured membrane results in two faces, implying two fracture planes, that is, one fracture plane located between the cytoplasm and the external cytoplasmic face of a membrane (concave fracture face) and the other fracture plane between the outside of a membrane and its surroundings (convex fracture face). When I started with freeze-fracturing, I adopted the above interpretation and I remember how difficult (but exciting) it was to understand the novel images. There was no reference point. However, the interpretation of Moor and Mühlethaler (1963) was challenged by Daniel Branton (Branton, 1966 [12]) by providing evidence that, regarding membranes, there is only one fracture plane, which splits the membranes’ hydrophobic interior. In other words, the convex and concave appearance are from one and the same fracture. It took some time before the vision of Branton became accepted, partly, because it was an opinion that differed from that of the authority, that is, the inventors of the technique.

What about bacteria? I delved into the topic by two approaches. Firstly, I made complementary replicas in such a way that I retained the fractured material, and made replicas of the original and of the material normally thrown away. To make a long story short, I obtained two replicas, each on its own electron microscopic grid, of one and the same fractured bacterium. How to find them?

**Figure 3:**
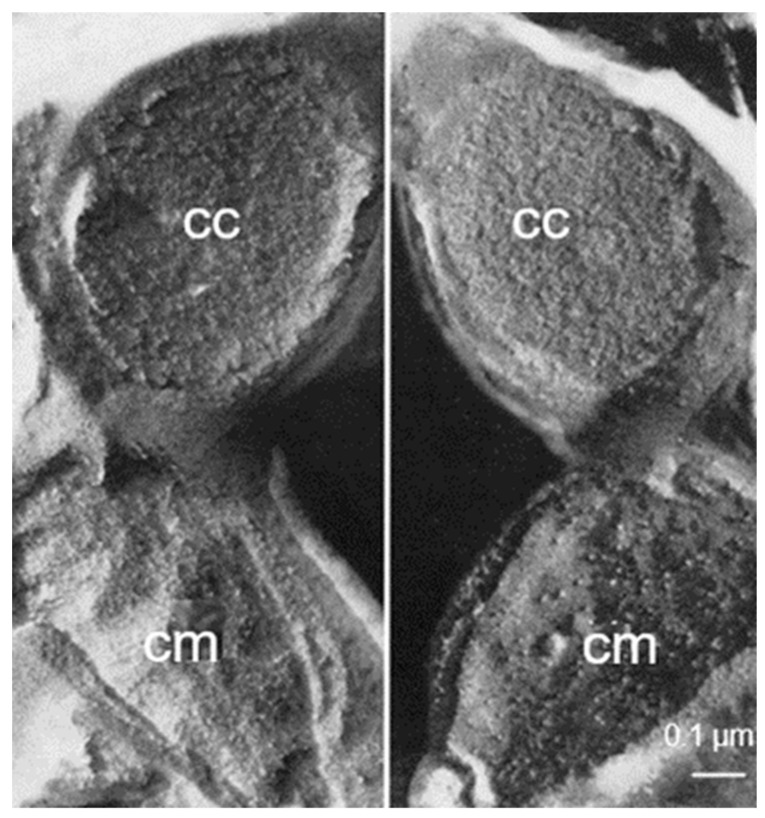
Complementary replicas of freeze-fractured *B. subtilis*. Note the convex and concave surfaces of the cell membrane (cm) at the lower left and the lower right, respectively. Above, the cell content (cc) has been cross-fractured with no clear-cut distinction of the nucleoid. Bar, 0.1 µm. Source: Modified Figure 1a,b from [13]. © 1971, Nanninga, N., Originally published in Journal of Cell Biology [13].

In the course of time, this turned out to be possible in a matter of minutes, though it did not, the first time. I take the liberty to recall this first attempt. Firstly, each grid with a replica was photographed in the electron microscope. Secondly, the two grids were printed, and the prints were placed and arranged on the floor of the library. Each grid had a surface of about 10 m^2^ (bacteria are small). I walked on them by foot with a pointing stick in my hand. It took some time before I found one and the same bacterium on the two different replicas (Figure 3; Nanninga, 1971 [13]). Another approach was to freeze-fracture fixed cells and subsequently embed them after thawing to be followed by thin sectioning. In this way, I could study fractured cells and inspect the fractures by classical electron microscopy. These results confirmed the interpretation of Branton (1966) [12].

In addition, I revisited *E. coli* with the late August van Gool and offered a different interpretation of the fracture faces (van Gool and Nanninga, 1971 [4]). What the membranous fracture faces demonstrated in particular was the asymmetric appearance of the cell membrane and the outer membrane, thus emphasizing the dramatic differences in the chemical compositions of the respective membrane faces.

## 3. Mechanism of DNA Segregation

### 3.1. DNA–Membrane Attachment and Envelope Growth

**Figure 4:**
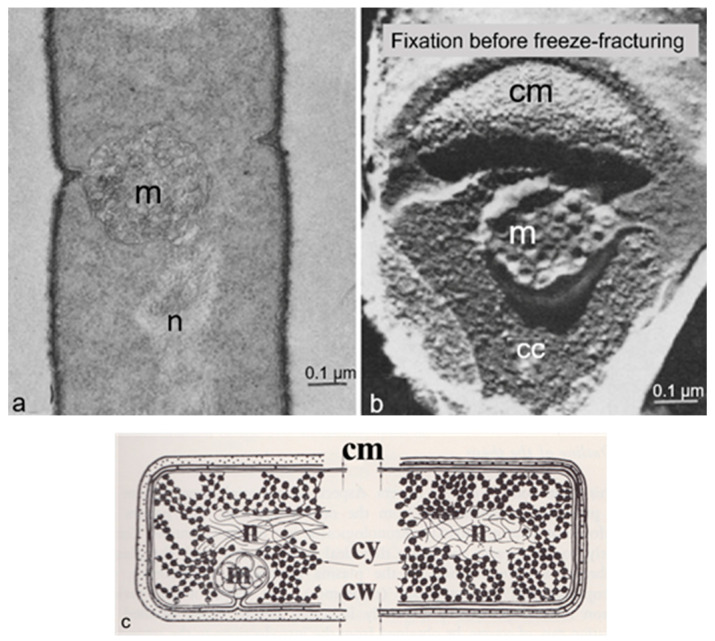
Thin section after RK-fixation of *B*. *subtilis* (**a**) with mesosome (m) and nucleoid (n). The mesosome is in contact with the nucleoid. To the right (**b**), a freeze-fracture image after chemical fixation. cc, cell content; cm, cell membrane; m, mesosome. Bar, 0.1 µm. Source: Modified Figures 2 (left) and 3 (right) from [15] © 1971, Nanninga, N., Originally published in Journal of Cell Biology [15]. (**c**) Schematic representation of the structures observed after sectioning of chemically fixed bacteria. The major components of a Gram-positive (*B. subtilis*; left), and a Gram-negative (*E. coli*; right) bacterium are shown. cm, cell membrane; cw, cell wall; cy, cytoplasm; m, mesosome; n, nucleoplasm. The cytoplasm is depicted as a network of ribosomes. In these prokaryotic cells, nucleoplasm and cytoplasm are not sharply separated from each other. Note the multilayered structure of the *E. coli* cell wall. The diameter of these bacteria is about 600 nm. Source: My thesis in 1970 [2].

A seminal paper also inspired by structures visible in the electron microscope dealt with “the regulation of DNA replication in bacteria” (Jacob et al., 1963 [14]). In early electron micrographs, DNA-containing regions could be recognized by the presence of a clew composed of more or less parallel electron-dense threads (presumably, coagulated DNA strands). The clews were largely located in the cell center and not surrounded by a membrane, hence the term nucleoid (nucleus-like). Extensive internal membranous structures could be seen in contact with the nucleoid in *Bacillus subtilis*, a Gram-positive bacterium. These structures seemed to arise from the enveloping cell membrane (Figure 4a; [15]) and they fed the notion that DNA is attached to a membrane. It was not a big step to connect DNA replication and segregation with envelope growth. In fact, DNA–membrane attachment became a popular research theme for decades, as witnessed by countless research papers.

### 3.2. The Jacob Model

**Figure 5:**
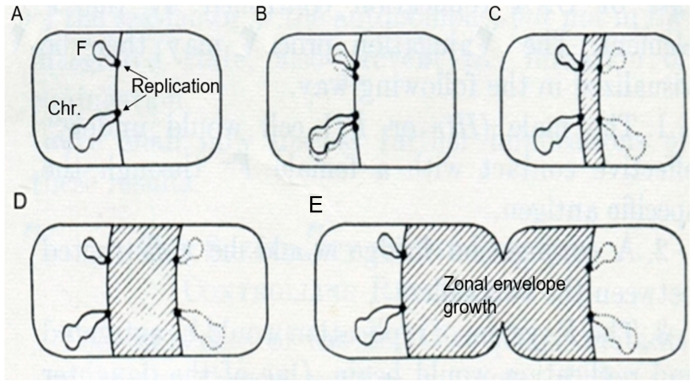
Model for the replication and subsequent segregation of a bacterial chromosome (Chr.) and an F factor (F). The circular molecules are membrane-attached, as is the replication apparatus. Replication takes place before segregation (**A**,**B**). Segregation occurs through zonal envelope growth (hatched areas in (**C**–**E**)). Source: Adapted Figure 10 from [14].

I have discussed the model proposed by Jacob et al. (1963) [14] before, under the heading of “Pictures Considered #32: A Model for DNA Segregation in 1963” in Elio Schaechter’s blog Small Things Considered (29 November 2015). I will follow the description of the model (Figure 5) as elucidated in the above contribution. In A of Figure 5, two circular replicons, the bacterial chromosome (Chr.) and a plasmid (F), are attached to “the equatorial perimeter”, which represents the “unit of segregation”. The circularity was deduced in genetic experiments from the linear sequences of genes in overlapping DNA fragments and by lightmicroscopic autoradiography (Cairns, 1963 [16]). In B, the “cell surface is assumed to transmit to the replicons the signal initiating their replication”. In C, D, and E, “Elements of the bacterial membrane are assumed to grow between the two planes of attachment of the daughter replicons putting them progressively apart”. (I have shortened the original legend, and wordings of the text of the paper of Jacob et al., 1963 [14] are between quotation marks).

For the present purpose, I will select three aspects of the model. Firstly, DNA is membrane-attached throughout the cell cycle. Secondly, DNA replication has been completed before segregation (B), in line with eukaryotic mitosis, where already-replicated chromosomes become separated. Thirdly, DNA segregation (C, D, E) is carried out by zonal growth of the cell envelope. Thus, the growing cell envelope with DNA attached to it would serve as an equivalent of the mitotic apparatus. As a matter of course, I should also mention here the pioneering work of Y. Hirota on bacterial thermosensitive mutants of DNA replication and of cell division (Hirota et al., 1968 [17]). He contributed to the development of the field of molecular genetics regarding the above topics.

### 3.3. DNA–Membrane Attachment

**Figure 6:**
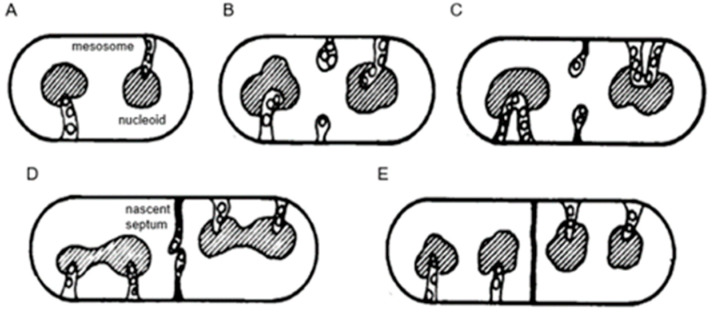
Model with the nucleoid attached to mesosomes as based on serial sectioning. Nucleoids divide, as do the mesosomes and septum arises with the help of mesosomes. (**A**) Mesosomes attached to nucleoids, (**B**) Septum formation with mesosomes, (**C**) Mesosome division and nucleoid growth, (**D**) Nucleoid and mesosome segregation, (**E**) Completion of nucleoid segregation enabled by mesosomes. Source: Modified Figure 23 [18]. Copyright © American Society for Microbiology.

An important consideration leading to the role of membranes in DNA segregation was the observation that the nucleoids in the Gram-positive *B. subtilis* appeared to be in contact with mesosomes (Figure 4a). Serial sections of *B. subtilis* for electron microscopy by Antoinette Ryter led to a model where mesosomes seem to mediate attachment of nucleoids to the cell membrane (A in Figure 6). Moreover, mesosomes divide in two, enabling separation of duplicated nucleoids (C, D and E). It should be noted that *E. coli* does not possess *B. subtilis*-like mesosomes, suggesting that its nucleoid is directly attached to the cell membrane (Ryter et al., 1968 [18]).

In 1963, the term membrane (cell membrane, cytoplasmic membrane, plasma membrane) had not the same meaning as it did in 1972, when the fluid mosaic model (FMM) was conceived (Singer and Nicolson, 1972 [19]). Similarly, there was not a consistent term for the nucleoid (nucleoplasm, bacterial chromosome, nucleus). Regarding the separation of nucleoids, the fluidity of the bacterial cell membrane precluded a conceptual function of the latter in this process. In the course of time, the focus shifted to the combination of cell membrane and peptidoglycan or murein layer (sacculus). In particular, the growing sacculus was seen as a component instrumental in cell elongation and, therefore, as an agent in DNA segregation. However, I will first return to the presumed role of mesosomes (Figure 4a).

### 3.4. Mesosomes

Around 1967, I started to use the freeze-fracture technique to study the ultrastructure of *B. subtilis.* Eventually, I came to the conclusion that mesosomes could not be shown in freeze-fractured cells. However, mesosomes became visible after freeze-fracturing when cells were first fixed with osmium tetroxide (R-K fixation) before freezing (Figure 4b), implying that mesosomes might be artifacts of preparation (Nanninga, 1969 [20]; Nanninga, 1971 [15]; Nanninga, 1973 [21]). I quote here the last sentence of my 1971 paper: “Finally, it can be said that some care is needed in drawing conclusions concerning the structure of mesosomes in chemically fixed material” (Nanninga, 1971 [19]).

**Figure 7:**
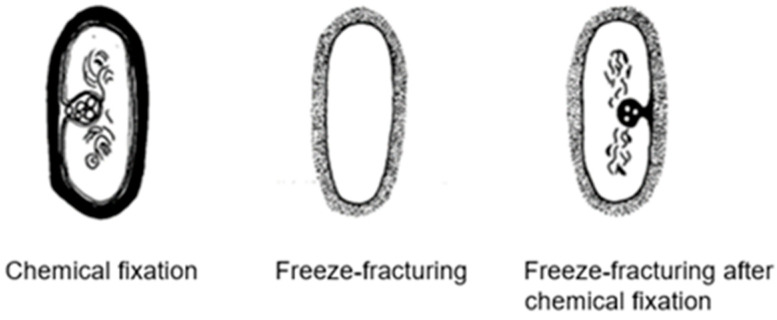
Diagram to depict the effect of chemical fixation on the appearance of mesosomes after freeze-fracturing. Mesosomes are not visible after freeze-fracturing without fixation. Source: Adapted Figure 7 from [20]. © 1971, Nanninga, N., Originally published in Journal of Cell Biology [20].

Presenting my observations on conferences produced disbelief and sometimes outspoken hostility. I learned that rationality and science do not always go together. My final sentence quoted above was an attempt to adopt a more or less neutral position (while softening my real opinion), though the accompanying published scheme left no doubt (Figure 7). For a more detailed discussion on freeze-fracturing of mesosomes, see Nanninga (1973) [21]. Extensive electron microscopic and biochemical experiments on the variability of mesosome structure after chemical fixation have been carried out by Silva et al. (1976) [22]. In the case of the nucleoid, lack of its clear-cut discernibility was not due to its absence (Nanninga, 1969 [19]). I will elaborate on this later on.

However, quite unexpectedly, mesosomes became fashionable again from the early nineties of the previous century onwards. They ended up in the field of epistemology. Some paper titles: “Facts, artifacts, and mesosomes: Practicing epistemology with the electron microscope” (Rasmussen, 1993 [23]), “Mesosomes and scientific methodology” (Hudson, 2003 [24]) and “The very reproducible (but illusory) mesosome” (Allchin, 2022 [25]). These are only a few examples. I found out that epistemologists disagree about thinking about science. Fortunately, one of them concluded that I was a real scientist: “For example, referring again to the mesosome episode, if I were to suggest that either Chapman or Hillier or Fitz-James or Nanninga is not in fact a scientist, then I would have a difficult time making my case “ (Hudson, 2003 [24]).

### 3.5. Envelope Structure

In the foregoing, I referred to the Gram-positive cell envelope of *B. subtilis,* i.e., the cell membrane and its attached relatively thick cell wall composed of peptidoglycan and teichoic acids. This was the image visible in the electron microscope after thin sectioning (Figure 4a). By contrast, the envelope of the Gram-negative *E. coli* proved quite different, revealing a more layered appearance after thin sectioning (de Petris, 1967 [26]; Figure 4c). In a pioneering study, Weidel et al. (1960) [27] had already isolated from *E. coli* B what has been termed the sacculus. Its isolation required various chemical treatments resulting most notably in a covalently linked macromolecule in the shape of the original bacterium. The sacculus had been visualized by shadowcasting. The envelope, as already mentioned above, revealed a layered appearance after thin sectioning (de Petris, 1967 [26]).

De Petris also employed various enzymatic and chemical treatments of intact cells and their fragments. Though using a different terminology, de Petris contributed to the concept of a three-layered structure of the *E. coli* cell envelope: outer membrane, peptidoglycan layer, and cell membrane (though the periplasmic space acquired a special consideration because the size of its volume appeared controversial (Graham et al., 1991 [28])).

## 4. Size Distributions by Electron Microscopy

### 4.1. Steady-State Growth and Agar Filtration Technique

Our change of thinking, already referred to above, from the static to the dynamic aspects of bacterial structure involved a shift from the individual cell as such to the individuals that are members of a population. What is needed, we (Conrad Woldringh and myself) realized, is not a random collection of cells, but a population with reproducible parameters in time. One such parameter is cell length, which one wishes to correlate with a cell cycle event in time. It dawned on us that an exponentially growing bacterial culture is not well defined and that steady-state growth is required (Maaloe and Kjeldgaard, 1966 [29]). In practice, to create steady-state growth, a small inoculum is used to start a batch culture, after which periodical dilutions with prewarmed growth medium are carried out to achieve a continuous exponential growth (cf. Fishov et al., 1995 [30] and references therein).

An important method to visualize populations was the agar filtration technique (Kellenberger and Arber, 1957 [31]). It has been widely used to make length distributions of bacterial cultures for electron microscopy and to screen for appropriate markers. In addition, it is easy to distinguish dividing from non-dividing cells. Methodically, the cells are fixed with 1% osmium tetroxide to a final concentration of 0.1%. The fixed cells are then applied to an agar filter and processed according to the filtration technique (Kellenberger and Arber, 1957 [31]). Cell length is measured on electron micrographs. In this way, steady-state culture conditions are combined with electron microscopy. (In steady-state, the measured length distributions are constant in time). Below follows an early example that refers to nucleoid separation in *E. coli* (Woldringh, 1976 [32]).

### 4.2. Length Distributions

**Figure 8:**
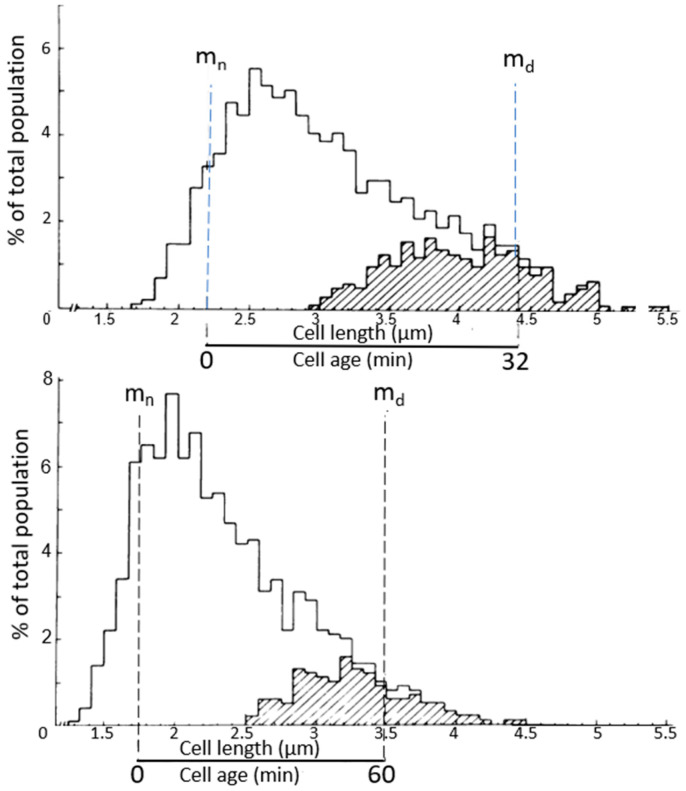
Length distribution of OsO_4_-fixed cells prepared by agar filtration. Above, *E. coli* B/r growing with a doubling time of 32 min. Below, doubling time 60 min. Hatched areas: Length distribution of cells in the process of constriction. Mean length of newborn (m_n_) and dividing cells (m_d_). Source: Figure 1 of [32]. Copyright © American Society for Microbiology.

In Figure 8, two *E. coli* length distributions are shown, wherein the dividing cells are indicated by hatched areas. The mean length of dividing cells (m_d_) can be calculated from the relationship m_d_ = ½ 1_max_ + 1_min_, as deduced by Harvey et al. (1967) [33]. The minimal cell length (l_min_) and the maximal cell length (l_max_) are taken from the length distribution. The mean length of the newborn cells (m_n_) = 1/2 m_d_. The distance between m_n_ and m_d_ represents the duration of the doubling time of the steady state cultures of 32 and 60 min, respectively. It is generally assumed that the relationship between length and age is exponential (for discussion, see Koppes and Nanninga, 1980 [34]). The technique has been helpful in establishing the timing of nucleoid separation in thin-sectioned cells, as described below. Though we did focus on cell length, cell diameter can also be interesting. The latter has been previewed by Zaritsky in 1975 [35] and studied extensively by Trueba and Woldringh (1980) [36].

### 4.3. Measuring Techniques

An important asset for us was the presence and dedication of Norbert O. E. Vischer for helping with and developing measuring tools for analyzing images of bacteria. One example is the software program ObjectJ, which he modified and improved for decades. Details are in the references: (Vischer et al., 1994 [37]; Vischer et al., 2015 [38]). The recent tool Coli-Inspector allowed the presentation of maps of fluorescent profiles of thousands of individually labeled cells. An example and technical details can be found in Figure 27 to be discussed later. The combination of bacterial physiology (steady-state), electron microscopy, and image analysis has been termed the Amsterdam School by Arthur Koch on several occasions. Its scientific context has been elaborated on by Arieh Zaritsky in a personal perspective on chromosome replication, cell growth, division, and shape (Zaritsky and Woldringh, 2015 [39]). Since my retirement, Tanneke den Blaauwen has continued and improved our work with the fullest dedication.

## 5. DNA Replication and DNA Segregation Go Hand in Hand

### 5.1. Nucleoid Size and Cell Length

In the model of Jacob et al. (1963 [14]; Figure 5), DNA segregation follows after the completion of DNA replication. The question arose, as mentioned before, whether this notion could be corroborated by electron microscopy. With this in mind, Woldringh (1976) [32] studied the morphological appearance of the nucleoid (or nucleoplasm) during cell elongation, i.e., from birth to division. For this purpose, he followed serially sectioned *E. coli* B/r A cells classified according to length. To allow an estimate of the relation between cell length and cell age, length distributions were made with the agar filtration technique of steady-state-grown cells. B/r strains were employed that had previously been used by Cooper and Helmstetter (1968) [40] to determine the timing of termination of DNA replication in synchronized cells. In this way, the cell length at which DNA replication terminates in the length distributions could be inferred. Visible separation of the nucleoids in Woldringh’s images appeared to coincide with the termination of DNA replication, as previously determined by Cooper and Helmstetter (1968) [40]. Before termination of DNA replication, the area occupied by the nucleoplasm in the sectioned cells increased in size with cell length. Earlier work by Cooper and Helmstetter (1968) [40] and Helmstetter and Cooper (1968) [41], complemented by the morphological studies of Woldringh (1976) [32], indicated that DNA replication and DNA segregation go hand in hand. If so, it means that classical eukaryotic mitosis does not apply to *E. coli* and presumably not to prokaryotic cells in general. So, how should prokaryotes and eukaryotes be compared? I have dealt with this topic separately elsewhere (Nanninga, 2001 [42]).

### 5.2. A Course in Electron Microscopy

**Figure 9:**
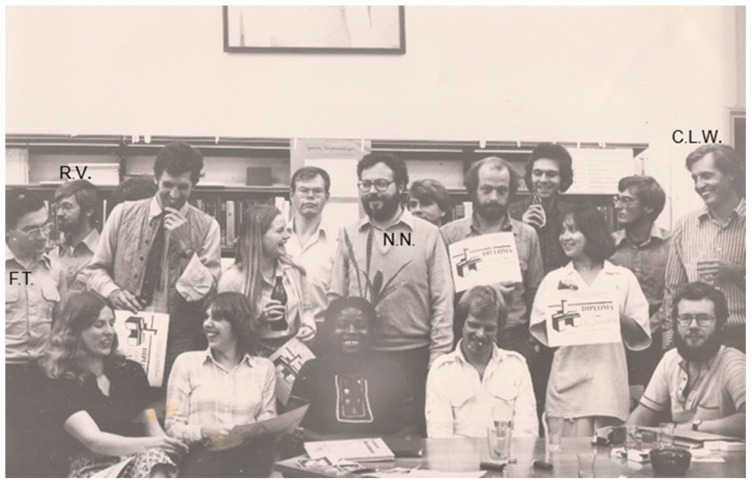
Diploma awarding after a Course in Electron Microscopy (1978). From left to right: F.T, Frank Trueba; R.V., Ronald Verwer; N.N., Nanne Nanninga; C.L.W., Conrad Woldringh.

In the meantime, we obtained obligations in teaching. One example was an introductory course in electron microscopy. The course was very popular and always attracted a very diverse population of all ages from Amsterdam. The included photograph is from 1978 and depicts a final stage: diplomas have been awarded (Figure 9). Two familiar persons at the left are Frank Trueba and Ronald Verwer. Also present are Conrad Woldringh (to the far right) and the writer of this article.

## 6. Peptidoglycan Assembly, Arrangement of Glycan Chains, and the Thickness of the Sacculus

### 6.1. Peptidoglycan Assembly

**Figure 10:**
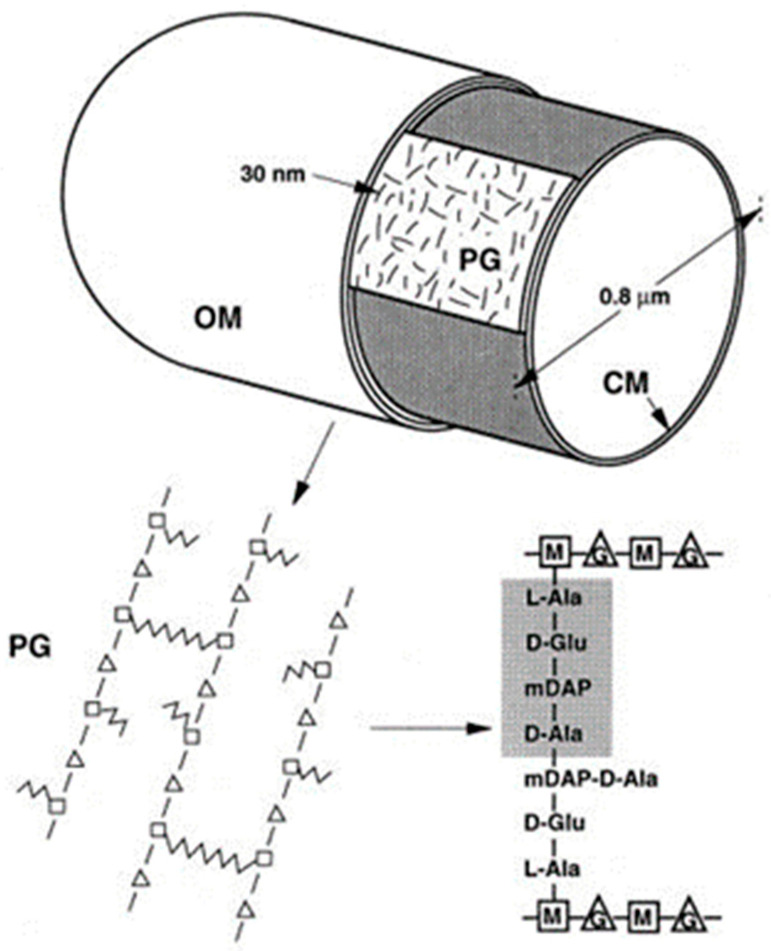
Arrangement of glycan chains in the peptidoglycan layer, the schematic structure of peptidoglycan, and the making of a cross-link. CM, cytoplasmic membrane; G, *N*-acetyl-glucosamine; OM, outer membrane; PG, peptidoglycan. Source: Figure 2 of [44]. Copyright © American Society for Microbiology and Figure 4 of Nanninga, N. et al., 1992. Symp. Soc. Gen. Microbiol. 47: 185–221. Copyright © Cambridge University Press.

In the early sixties of the previous century, the peptidoglycan layer (murein layer or sacculus) was isolated from *E. coli* (Weidel et al., 1960 [27]; Weidel and Pelzer, 1964 [43]). In the electron microsope, as mentioned already above, it was visible as a flattened structure with the outline of the bacterium it came from. The sacculus, which was supposed to maintain cell shape, is composed of glycan chains interconnected with short peptide chains. It is a covalent structure, and as such, it represents a single macromolecule (Nanninga, 1998 [44] and references therein; Figure 10).

**Figure 11:**
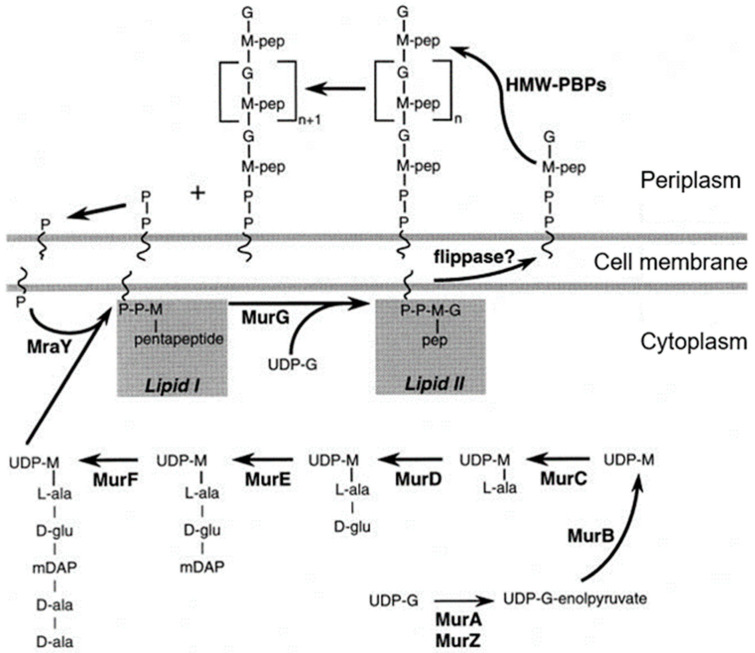
Peptidoglycan assembly. The cytoplasmic steps leading to the prenylated pentapeptide (lipid II) are shown. Through the action of a hypothetical flippase, the disaccharide pentapeptide becomes exposed to the periplasm. The membrane-bound disaccharide peptide becomes attached to a polymer that is also membrane-bound. Glycan chain elongation occurs by transglycosylation. Elongation can occur at the nonreducing end of the glycan chain (this figure) or at the reducing end (not shown in this figure). G, *N*-acetylglucosamine; M, *N*-acetylmuramic acid; P, undecaprenylphosphate; PP, undecaprenyl biphosphate; pep, pentapeptide. Source: Figure 3 of [44]. Copyright © American Society for Microbiology.

Its assembly begins in the cytoplasm, is continued by membrane proteins (in particular PBPs), and is completed as a constituent of the cell envelope (Nanninga, 1998 [44]; Figure 11). Briefly, UDP-MurNAc-pentapeptide in the cytoplasm is bound to undecaprenyl phosphate in the cell membrane by MraY (a translocase) creating lipid I. MraY contains many membrane spanning sequences. Lipid II is made by adding lipid I to UDP-Glc-NAc by MurG (a transferase). MurG is located at the cytoplasmic side of the cell membrane. Next, the prenylated disaccharide pentapeptide has to be presented to the periplasm by a flippase. In 1998, its identity was not yet known (see further on). In the periplasm, the main actors are the penicillin-binding proteins (PBPs; Spratt, 1975 [45]). Here, I will not give further particulars.

The peptidoglycan layer, though a single macromolecule, is not a static structure. Dynamics include rearrangements, turnover, recycling, degradation, and autolysis (van Heijenoort, 1992; personal communication). For more extensive information on peptidoglycan assembly at the end of the nineties of the previous century, see van Heijenoort, 1996 [46] and Höltje (1998) [47]. For a recent excellent review, cf. Egan et al., 2020 [48].

Finally, note that many genes relevant for peptidoglycan synthesis and cell division are located in the 2-min region of the *E. coli* chromosome, which leads to the question of how and to what extent their expression is coordinated (Rothfield and Garcia-Lara, 1996 [49]). This will be elaborated later on.

### 6.2. Arrangement of Glycan Chains

In collaboration with the group of the late Uli Schwarz in Tübingen, we isolated purified sacculi from *E*. *coli* and incubated them with enzymes that disrupt peptidoglycan. Thereafter, they were prepared for electron microscopy. Sacculi became fully disrupted after incubation with *E. coli* transglycosylase or egg white lysozyme. These enzymes hydrolyze the glycan chains. However, after hydrolysis of the peptide bridges which link the glycan chains with an *E. coli* endopeptidase, a preferential orientation of the remaining glycan chains more or less perpendicular to the length axis of the cell was observed. This suggests that cell elongation involves insertion of new glycan chains in the same orientation, thereby keeping cell diameter constant (Verwer et al., 1978 [50]).

### 6.3. Thickness of the Peptidoglycan Layer

In a later stage, there reemerged the thickness of the peptidoglycan layer (Wientjes et al., 1991 [51]), in particular, through the “make before break” concept of the late Arthur Koch (1984) [52]. That is, growth of a single-layered covalently closed peptidoglycan should not lead to rupture. Hence, new glycan chains are first cross-linked to existing peptidoglycan before old cross-links are broken. Next, the new chains are pulled into the stress-bearing plane of the peptidoglycan by osmotic pressure (Koch, 1984 [52], Höltje, 1998 [47]). I also would like to acknowledge here our fruitful contacts with the late Jochen Höltje and coworkers, the successor of Uli Schwarz in Tübingen.

In the meantime, the sacculus grew thicker and thicker (I will refrain from references), so we thought it appropriate to attempt to measure its thickness again. Firstly, a radiochemical method, as outlined by Wientjes et al. (1991) [51], was employed. This method is based on the steady-state incorporation of [meso-^3^H] diaminopimelic acid ([^3^H] Dap) during several generations. One can calculate the number of Dap molecules per sacculus from the cell concentration and the specific activity of the [^3^H] Dap. Secondly, one can measure the Dap content chemically in sacculi isolated from a known number of cells. With both methods, a value of about 3.5 × 10^6^ Dap molecules per sacculus was obtained. Combined with electron microscopic measurements of the surface area of the cells, the data indicate an average surface area per disaccharide unit of ca. 2.5 nm^2^. This suggests again that the peptidoglycan is organized in a monolayered structure.

However, there remained the question of how the peptidoglycan layer is growing. The answer has been sought by application of electron microscopic autoradiography of cells pulse-labeled with [^3^H] Dap. We measured the position of silver grains on electron microscopic images of non-dividing and dividing cells.

## 7. Zonal or Diffuse Growth of the Peptidoglycan Layer?

Early pioneering autoradiographic experiments on the insertion of [^3^H] Dap into peptidoglycan in *E. coli* seemed to show the expected central zone of envelope growth in the electron microscope (Schwarz et al., 1975 [53]). However, diffuse incorporation in the lateral wall was also observed. From several subsequent autoradiographic experiments, it became clear that insertion of labeled [^3^H] Dap in elongating cells occurs diffusely over the entire cell envelope (Verwer and Nanninga, 1980 [54]; Burman et al., 1983 [55]; Woldringh et al., 1987 [56]; Wientjes and Nanninga, 1989 [57]). By contrast, sharply incorporated [^3^H] Dap could be seen in constricting cells. These experiments thus indicated that envelope growth in between the postulated DNA–membrane attachment points (zonal growth in Figure 5) does not function as part of a prokaryotic equivalent of a mitotic apparatus. So, how are replicated nucleoids segregated without help from the cell envelope and how does replication take place, that is, before or during segregation? These problems have been addressed by Conrad Woldringh (Woldringh, 2023 [58]).

## 8. Zonal or Diffuse Growth of the Outer Membrane?

With respect to the outer membrane, there was the same discussion as with the peptidoglycan layer. Is the insertion of outer membrane proteins during elongation zonal or diffuse? We were fortunate, being able to collaborate with the late inspiring Bernard Witholt and coworkers of the University of Groningen. We could combine his experience with the induction of outer membrane proteins of *E. coli* with our electron microscopic analytics. Induction was roughly for about 5 min and therefore comparable to a radioactive pulse. Thus, we embarked on the topography of LamB (Vos-Scheperkeuter et al., 1984 [59]) and of lipoprotein (Lpp; Hiemstra et al., 1987 [60]).

### 8.1. Induction of LamB

LambB is an outer membrane component (a transmembrane porin) as well as a phage λ receptor. Wild-type cells were induced with maltose and cyclic-AMP and a lac-lamB fusion strain with isopropyl-ß-D-thiogalactopyranoside (IPTG). Gold-labeled antibodies were bound to LamB proteins exposed at the surface and their topography analyzed. It was concluded that insertion of LamB proteins into the cell envelope occurred at multiple sites over the entire cell surface, and also at the site of constriction.

### 8.2. Induction of Lipoprotein (Lpp)

Lpp, which is covalently linked to the peptidoglycan layer (Braun and Rehn, 1969 [61]), is not exposed at the cell surface, thus prohibiting its direct labeling. Therefore, we had to treat cells with Tris-EDTA to make the Lpp accessible for labeling with a protein-A gold probe. Lpp was briefly induced with IPTG in cells carrying lac-lpp on a low-copy-number plasmid in an *E. coli lpp* host. In addition, in this case, the topography of Lpp was diffuse and not zonal, and at the constriction site [60].

For a more recent paper on LamB, see Gibbs et al. (2004) [62]. Sheng et al. (2023) [63] confirmed the tight localization of existing Lpp on the sacculus with atomic force microscopy, thus confirming earlier results with respect to the existing distribution of Lpp. The mode of insertion by induction was not carried out.

## 9. Centrifugal Elutriation and Peptidoglycan Synthesis in *E. coli*

In a later stage, we wished to compare [^3^H] Dap incorporation in dividing and non-dividing cells. For this, two approaches have been employed. It required methods to distinguish between the two populations. Firstly, we employed synchronized cells, which allowed the application of biochemical methods, and secondly, we employed electron microscopic autoradiography, which was applied to steady-state-grown cultures in the presence of [^3^H] Dap. In the latter case, we could easily identify dividing and non-dividing cells in the electron microscope.

### 9.1. Selection of Small Cells by Centrifugal Elutriation

To allow analysis of peptidoglycan synthesis during the cell cycle, one needs an enrichment procedure for dividing and non-dividing cells. A pioneering method for *E. coli* B/r strains was one where cells were attached by filtration to a membrane whereafter the latter was turned upside down and growth medium was poured through the filter. Under these conditions, the stuck cells continued their growth (and the division process) so that newborn cells left the filter specifically after cell separation. Newborn cells were collected in the cold to arrest their growth. Regrowth of the “babies” produced a synchronous culture (Helmstetter and Cummings, 1964 [64]; Helmstetter, 2023 [65]), invaluable for the experimental background of the Cooper-Helmstetter model (Cooper and Helmstetter, 1968 [40]). See also Helmstetter (2023) [65].

However, initially, not all *E. coli* strains appeared suitable for the above synchronization procedure, presumably because attachment to the membrane filter is influenced by the chemical composition of the *E. coli* cell wall and its appendages. In our case, we wished to study aspects of peptidoglycan synthesis during the cell cycle in *E. coli* by using specific K-12 (non-B/r) strains. This led us to search for an alternative cell separation method to allow synchronous growth. In collaboration with the late W. S. Bont of the Netherlands Cancer Institute (Amsterdam), we embarked on the adaptation of centrifugal elutriation for the separation of bacterial cells (Figdor et al., 1981 [66] and references therein). In centrifugal elutriation, controlled particle flow is in a direction opposite to that of sedimentation. Under appropriate conditions, the smallest particles (in our case, the smallest *E. coli* cells) can be washed out and collected. Elutriated cells were, before regrowth, collected in the cold as with the “baby machine”.

The above two cell separation methods are similar in the sense that small cells are selected to allow embarkment on synchronous growth. The selection criteria are, however, essentially different: age (baby machine) versus size (elutriation). Nevertheless, both approaches can produce separation of dividing and non-dividing cells.

### 9.2. Peptidoglycan Synthesis in Non-Dividing and Dividing Cells

Peptidoglycan synthesis was determined by following the incorporation of [^3^H] Dap by pulse-labeling of *E.coli* MC4100 *lysA*, either by using synchronized cultures (see above) or by autoradiography.

**Figure 12:**
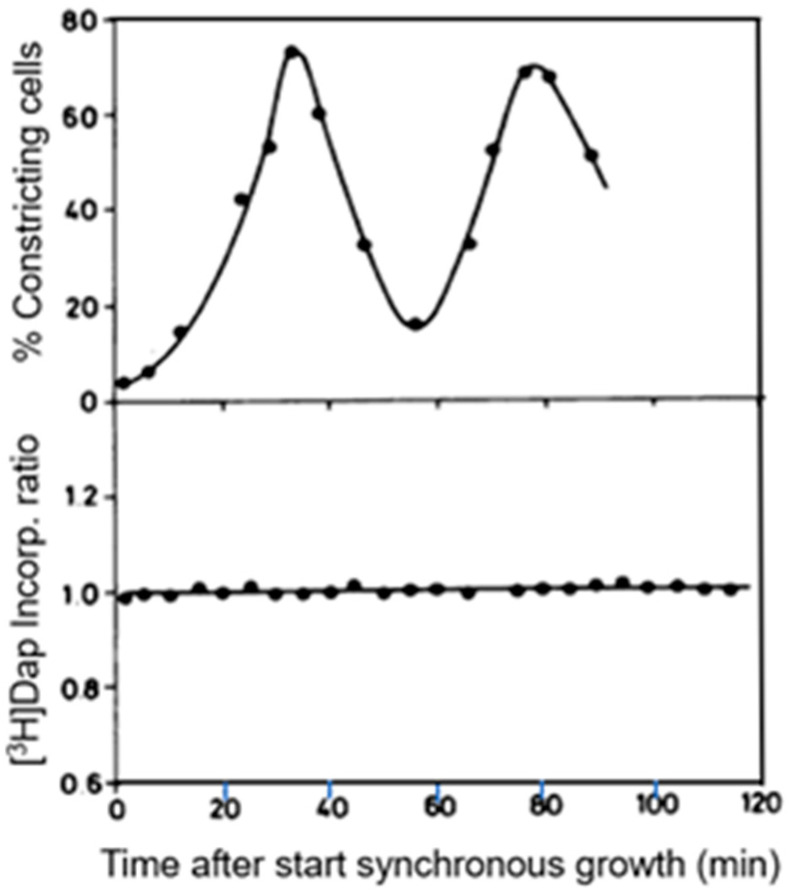
Percentages of cells showing a visible constriction after centrifugal elutriation, as judged by electron microscopy of whole-mount preparations (**top**). Pulse-labeling with [^3^H] Dap (**bottom**). The ratios of the pulse values of the synchronous and the control culture are given. For details, see [57]. Incorp., Incorporation. Source: Figure 1b,c from [57]. Copyright © American Society for Microbiology.

Small cells were separated by centrifugal elutriation and, as a control, asynchronous cultures were put through the elutriator as well (Creanor and Mitchison, 1982 [67]; Wientjes and Nanninga, 1989 [57]). Regrowth of the cooled samples at 37 °C was for two division cycles and the percentages of constricting cells varied between about 2 and 80% (Figure 12). The rate of [^3^H] Dap incorporation was measured in samples of synchronized and of exponentially growing cells. The incorporation ratios of the two types of cultures did not change during the experiment (Figure 12). This suggests that during the division cycle, no noticeable variation(s) in [^3^H] Dap incorporation occurs. On the other hand, it could not be excluded that a percentage of 80 for dividing cells might be too low to detect changes in incorporation.

**Figure 13:**
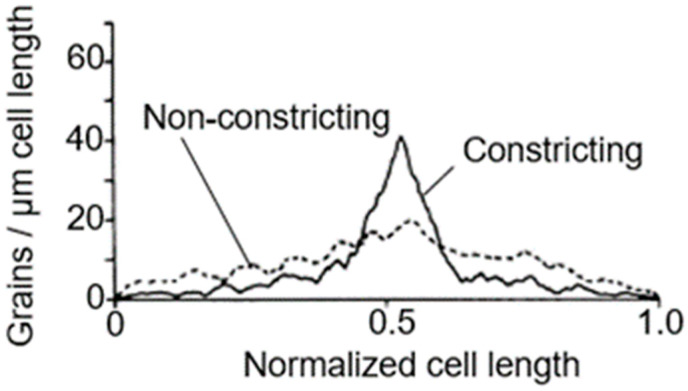
Comparison of the topography of [^3^H] Dap incorporation in constricting (-) and nonconstricting (--) cells of *E. coli* MC4100*lysA*. The grain distributions over the cells are plotted as grain densities (number of grains per micrometer of cell length) versus normalized cell length. For deeply constricting cells, 399 grains were counted on 23 cells and the average cell dimensions were 2.64 by 0.75 µm. The cells are positioned with their longest half to the left. For nonconstricting cells 867 grains were counted on 94 cells and the average cell dimensions were 1.72 by 0.75 µm. Source: Modified Figure 4 of [57]. Copyright © American Society for Microbiology.

To further assess this problem, we used again electron microscopic autoradiography on steady-state cultures of the same strain. As indicated before, electron microscopy allows an almost complete distinction of dividing and non-dividing subpopulations. It appeared that dividing cells of the same normalized length as the non-dividing ones produced more silver grains in the cell center (for details, see Wientjes and Nanninga, 1989 [57]). By comparing the topography of [^3^H] Dap incorporation in the two cases, it can be seen that incorporation at the site of division proceeds at the expense of lateral incorporation (Figure 13; uninterrupted line: dividing cells. Interrupted line: non-dividing cells). An additional interpretation of this figure can be found below.

**Figure 14:**
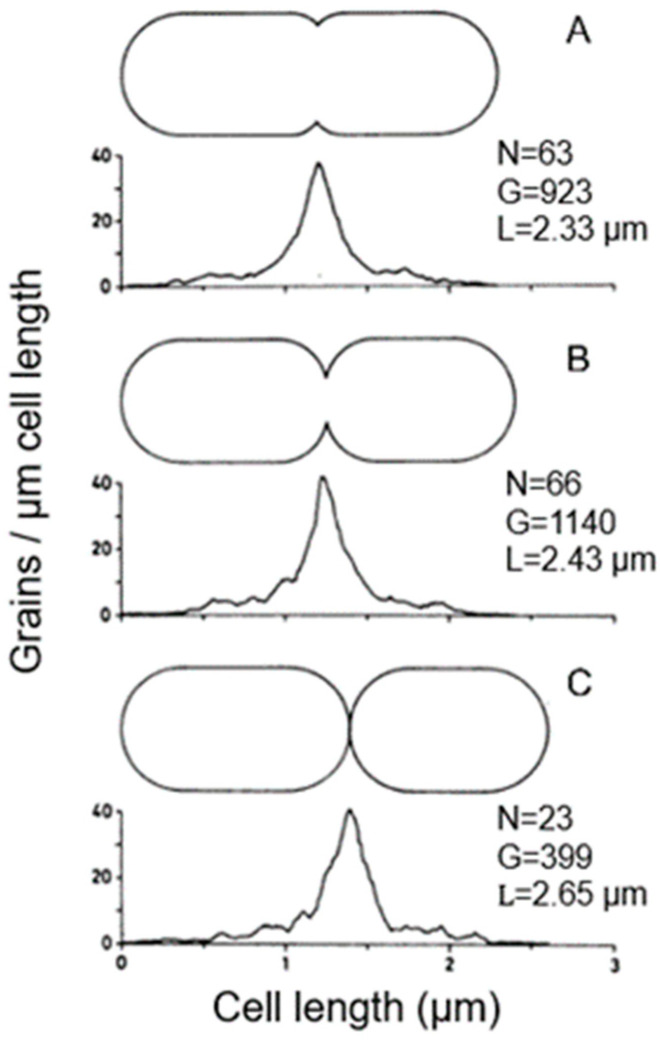
Silver grain distribution over cells in progressing stages of constriction (cells with slight (**A**), medium (**B**), and deep (**C**) constrictions). For labeling conditions, see Wientjes and Nanninga (1989). The grain distributions are plotted as grain densities versus cell length. The cells are positioned with the longest cell half to the left. Drawings of the cells of the different length classes are shown above the distributions, and the number of cells (*N*) and grains (*G*), as well as the average length of the cells (*L*), are given. Source: Modified Figure 5 [57]. Copyright © American Society for Microbiology.

Electron microscopy also allows the distinction of the topography of [^3^H] Dap incorporation in cells with slight, moderate, and deep constrictions (Figure 14). In these cases, the central peak did not widen during progression of constriction, implying that surface synthesis is not evenly distributed over the nascent polar cap. It suggested incorporation at the leading edge of the constriction. For this interpretation, we also considered that in plasmolyzed *Salmonella typhimurium* cells (similar to *E. coli*), the leading edge of constriction resisted separation of the various envelope layers (MacAlister et al., 1987 [68]). Presumably, this particular tightness at the leading edge reflects local peptidoglycan synthesis, as in Figure 14.

## 10. Further Details of Peptidoglycan Synthesis: Composition and Mode of Insertion

At the Department of Microbiology, the group of Jan T. M. Wouters acquired expertise in operating the chemostat under various conditions. One study pertained to the influence of various growth conditions on peptidoglycan structure as analyzed by high pressure liquid chromatography (HPLC; Driehuis and Wouters, 1987 [69]). Notably, it was found that the composition of the growth medium dominated cell shape with respect to peptidoglycan structure. Cell shape refers to long cells (relatively little polar cap material) and short cells (relatively much polar cap material).

However, it remained possible that chemical differences existed between the lateral wall and polar cap. So, we turned to peptidoglycan structure in synchronized cultures, asking what products were being formed after incorporation of [^3^H] Dap after pulslabeling. Table 1 gives an impression of what kind of peptidoglycan digestion products can be found in exponentially growing cells by HPLC (de Jonge et al., 1989 [70]). Note, the ubiquitous presence of Tet and Tet-Tet (bis-disaccharide tetratetra) compounds.

After applying the centrifugal elutriation procedure, fractions containing a high proportion of dividing cells were compared to those containing a high proportion of non-dividing cells by HPLC. No difference between the samples was found regarding the peptidoglycan peptides, indicating that division does not require a specific peptide. From the acceptor–donor radioactivity ratio (ADRR) in Tet-Tet, the mode of insertion was derived in dividing and non-dividing cells. In the former, the ratio was high, and in the latter, the ratio was low. This was interpreted to mean that, during cell elongation, the insertion was single-stranded and, during division, multi-stranded (“or sequential single-stranded”). The relationship between ADRR and crosslinking has been discussed by Cooper (1990) [71] and replied by Driehuis et al. (1991) [72], while emphasizing the difference between crosslinking and crosslinkage.

## 11. Presence of Penicillin-Binding Proteins (PBPs) in Synchronized Cells and Their Cell-Cycle-Dependent Activities

### 11.1. Presence of PBPs during the Cell Cycle

“Distinct penicillin binding proteins involved in the division, elongation, and shape of *Escherichia coli* K12”. This was the informative title given by Brian Spratt for the paper about the detection of penicillin-binding proteins (PBPs; Spratt, 1975 [45]). The PBPs were detected after labeling them with [^14^C] benzylpenicillin or [^14^C] mecillinam (FL1060) followed by cell fractionation. Subsequently, they became known as PBP3, PBP2, and PBP1, respectively. PBP3 and PBP2 are transpeptidases, PBP1, now PBP1A and PBP1B, are bifunctional and as such combine transpeptidase and glycosyltransferase activities. The PBPs play various roles in peptidoglycan biosynthesis. Here, I have referred to the main PBPs and I wish here to acknowledge the fruitful collaboration with Brian Spratt on several occasions. For a modern overview, cf. Sauvage and Terrak (2016) [73].

**Figure 15:**
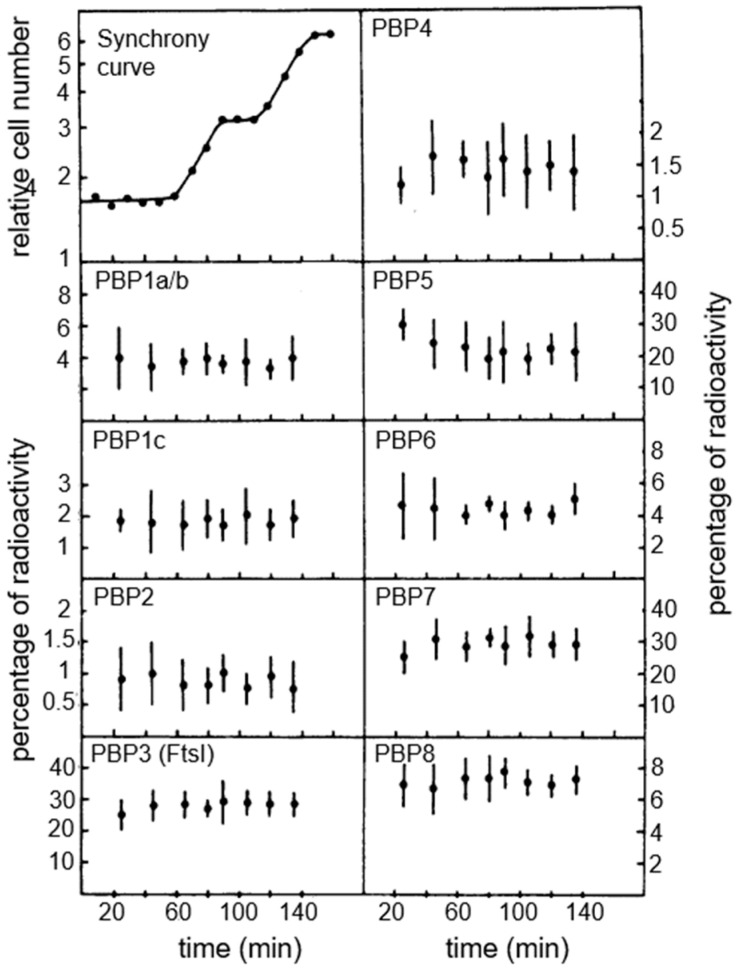
Relative intensities of PBPs after labeling of intact cells with [^125^I] ampicillin. Intact cells from synchronous cultures (centrifugal elutriation) were labeled with [^125^I] ampicillin, and the percentage of radioactivity in each PBP band was measured. Average values and standard deviations are shown. PBP1a/b, PBP1c, PBP2, PBP3 (FtsI), PBP$, PBP5, PBP6, PBP7, and PBP8 are indicated. Source: Modified Figure 2 of [74]. Copyright © American Society for Microbiology.

The availability of the centrifugal elutriation technique allowed us to study the binding of [^125^I] ampicillin to PBPs under various experimental/technical conditions of *E. coli* PA3092 (a K-12 derivative). The main result was that all PBPs became labeled to the same extent, in intact cells as well in membranes derived from them. Figure 15 shows the results with all PBPs (PBP1A/B. PBP1C, PBP2-PBP8) obtained from intact cells. Overall, they appear in a constant ratio during the division cycle, and I quote the general conclusion: “The *E. coli* cells exert their control on shape maintenance and cell wall growth apparently not on the level of concentration of PBPs in the cell but rather on activation of existing components” (Wientjes et al., 1983 [74]). So, what about “activation of existing components”?

### 11.2. Inhibition of PBP2 and PBP3 during the Cell Cycle

**Figure 16:**
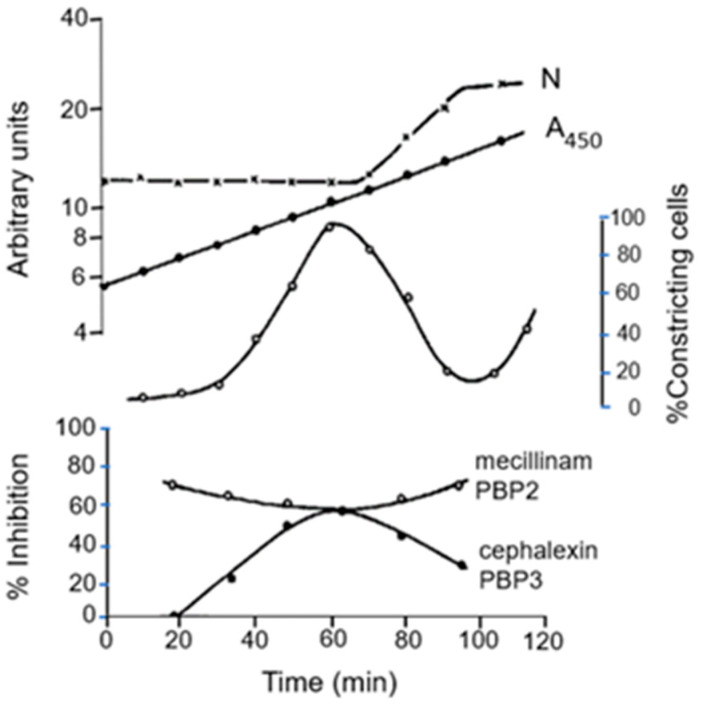
Contribution of PBP2 and PBP3 to peptidoglycan synthesis in relation to the division cycle. The cell number (N) and absorbance (A_450_) of the main culture are shown in the top panel, and the percentage of constricted cells in the middle panel. The lower panel shows the inhibition percentages of [^3^H] Dap incorporation in the subcultures by mecillinam and cephalexin (for details, see Wientjes and Nanninga, 1991 [75]). Source: Figure 4 of [75]. © Elsevier France.

We tried to answer this question by adding specific antibiotics to synchronized *E. coli* K-12 strain MC4100 *lysA* cells (Wientjes and Nanninga, 1991 [75]). We focused on cell elongation and cell division. In Figure 16, we can observe the rise and fall of the percentage of constricting cells after regrowth of selected small cells. The effect of the antibiotics mecillinam (PBP2) and of cephalexin (PBP3) is plotted as percentage of inhibition of [^3^H] Dap incorporation. It appears that PBP2 is active all the time (cell elongation), and PBP3 predominantly in dividing cells. Below, I will detail the cellular location of PBP2.

**Figure 17:**
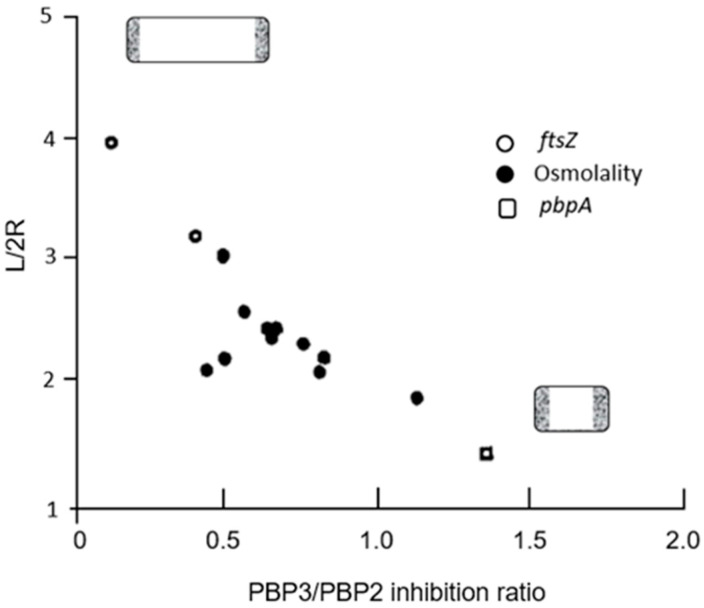
Relation between cell shape and PBP3/PBP2 inhibition ratio. *E. coli* MC4100*lysA* was grown in a phosphate-based minimal medium at 37 °C (doubling time ca. 45 min). The average cell dimensions were measured, and the length/width ratio (L/2R) was calculated. Inhibition of [^3^H] Dap incorporation was done with cephalexin (10 mm/mL) and by mecillinam (2 mm/mL). The ratio of these inhibition percentages were taken as thePBP3/PBP2 ratio. Further details concerning osmolality and *pbpA* and *ftzs* mutants can be found in [75]. Source: Modified Figure 6 of [75]. © Elsevier France.

The roles of PBP2 and PBP3 in cell elongation and cell division, respectively, also became clear by plotting the aspect ratio (L/2R) against the PBP3/PBP2 inhibition ratio. That is, the longer the cell, the smaller the inhibition ratio, and vice versa. For further details, see the legend of Figure 17 (Wientjes and Nanninga, 1991 [75]).

## 12. Localization of Cell Division Proteins

### 12.1. Making Monoclonal Antibodies

Being microscopists (at least in part), we wished to localize proteins of interest in *E. coli*. This plan was developed before the ubiquitous use of fluorescent markers and the use of green fluorescent proteins. In the latter case, the accompanying loss of resolution was compensated for by the prospect of visualizing fluorescent labels in living cells. It also implied a shift from electron microscopy to fluorescence microscopy (in some cases). However, the detection of FtsZ at the site of constriction by classic immunolabeling of thin sections for the electron microscope was quite exciting (Bi and Lutkenhaus, 1991 [76]), and it stimulated further our project, which started in 1989, to make monoclonal antibodies against cell division proteins. For this, we were happy to collaborate with Arend H. J. Kolk from the N. H. Swellengrebel Laboratory of Tropical Hygiene, Royal Tropical Institute, Amsterdam. Notably, FtsZ (Voskuil et al., 1994 [77]; Koppelman et al., 2004 [78]), PBP1B (den Blaauwen et al. (1989 [79], 1990a [80], 1990b [81]); Zijderveld et al. (1991 [82], 1995a [83], 1995b [84])) and FtsQ (Buddelmeijer et al., 1998 [85]) were studied. These studies involved, amongst others, epitope mappings to chart portions of the respective proteins. Here, I will specifically dwell on the data obtained with FtsQ because they could be placed in a somewhat wider context.

### 12.2. Location of FtsQ

We found a central location by immunofluorescence microscopy with specific monoclonal antibodies, as expected, though also in non-central positions. It appeared that the periplasmic part of FtsQ was required for location at the cell center (Buddelmeijer et al., 1998 [85]). Subsequently, it was found that FtsQ occurs in a complex with FtsL and FtsB and that the complex formed before entry into the divisome (Buddelmeijer and Beckwith, 2004 [86]). Further structural and mutational analysis of FtsQ revealed that divisomal positioning of FtsQ and its interaction with Fts L and FtsQ is dependent on two different periplasmic domains (van den Ent et al., 2008 [87]). The first domain is located near the cell membrane, the second domain near the C-terminus. “Both domains act together to accomplish the role of FtsQ in linking upstream and downstream cell division proteins within the divisome” (van den Ent et al., 2008 [87]). This emphasizes the organizational role of FtsQ in divisome construction.

However, “Noncentral FtsQ foci were found in the area of the cell where the nucleoid resides and were therefore assumed to represent sites where the FtsQ protein is synthesized and simultaneously inserted into the cytoplasmic membrane”. “An interesting speculation is that the foci might occur where the division and cell wall gene cluster (dcw cluster) resides. To corroborate this intriguing possibility, it will be necessary to combine gene and protein localization studies” (quoted from Buddelmeijer et al., 1998 [85]). And so we did (Roos et al., 2001 [88]).

### 12.3. Cellular Localization of Cell Division Genes (ftsQAZ Cluster)

**Figure 18:**
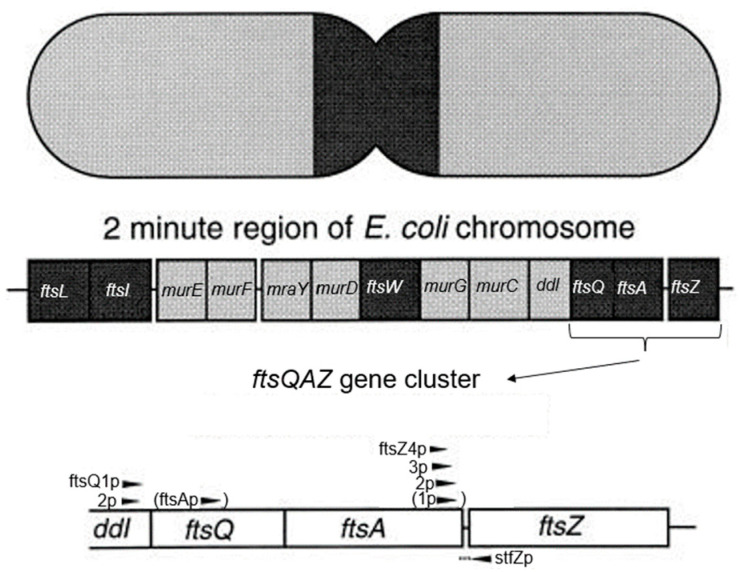
Clustering of genes involved in cell division and cell wall synthesis (dcw cluster) in the 2-min region of the *E. coli* chromosome. Cell division genes are darkly shaded. The genes involved in the production of prenylated disaccharide pentapeptide are lightly shaded (Cf. Figure 11). The *ftsQAZ* gene cluster is depicted at the bottom of the figure. Note the position of the various promoters (see also [90]). Source: Figure 4 of [44] and references therein. Copyright © American Society for Microbiology [44].

The *ftsQ* gene occurs in an *ftsQAZ* gene cluster in the 2-min region of the *E. coli* chromosome. The 2-min region encompasses numerous other genes related to cell division and cell wall synthesis (Wijsman [89]). Hence, as already mentioned above, it has been denoted as the dcw cluster (Figure 18; Ayala et al., 1994 [90]). For further details on dcw, cf. Rothfield and Garcia-Lara (1995 [49]) and Vicente et al. (1998 [91]). Since FtsQ is a transmembrane protein, it was to be expected that the nucleoid, as required by cotranscriptional-cotranslational insertion, is linked through mRNA and ribosomes to the cell membrane (Norris and Madsen, 1995 [92]; Woldringh, 2002 [93]).

A probably naïve supposition was our idea that division genes could be located near the cell center in the vicinity of the nascent divisome during their transcription. This possibility came to our mind before genes or gene regions could be demonstrated by microscopy, the underlying idea being that in bacteria gene and gene product are spatially close together. How to detect genes in the cell?

The microscopic detection of the origin of replication (*oriC*) inside the living cell by fusing gene regions in the neighbourhood of the origin with GFP represented a breakthrough. This was done for *E. coli* (Gordon et al., 1997 [94]) as well as for *B. subtilis* (Webb et al., 1997 [95]). Other genes followed. This took place more or less coincidently with our localization study of FtsQ. In the meantime, we had acquired practical experience (in another context) with fluorescence in situ hybridization (FISH). So, we decided to locate the *ftsQAZ* gene cluster by FISH during the cell cycle of *E. coli* (Roos et al., 2001 [88]). As reference points, we used *oriC* (near the origin) and *minB* (near the terminus). For all genes, plasmid probes were employed for hybridization.

**Figure 19:**
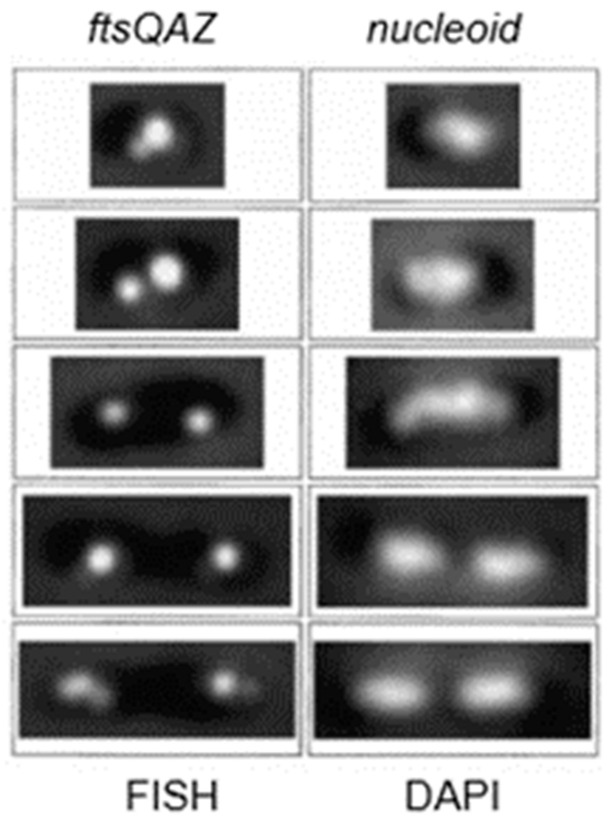
Hybridization foci of *ftsQAZ-* and DAPI-stained nucleoids in fixed cells of increasing length. Cells and foci were photographed in phase contrast and with an Alexa filter (**left**); cells and nucleoids were photographed in phase contrast and with a DAPI filter (**right**). Cells were hybridized with an *ftsQAZ* probe. Source: Modified Figure 2 of [88]. © John Wiley and Sons.

**Figure 20:**
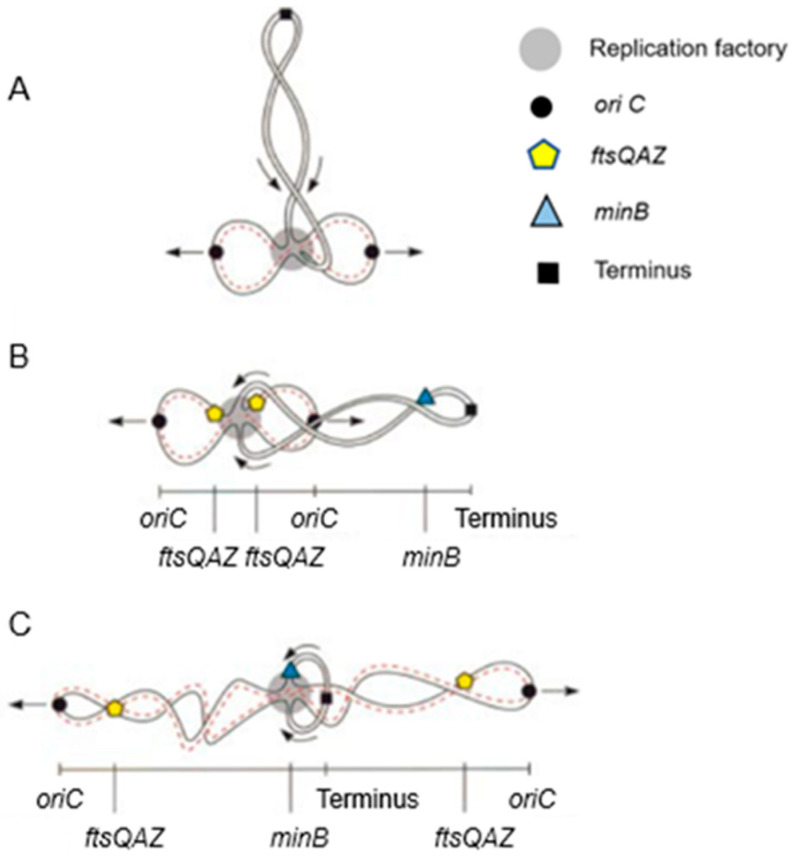
Model for sequential separation of DNA subregions on the *E. coli* chromosome. (**A**) redrawing of the original Dingman figure with the terminus perpendicular to the fixed replisome (replication factory), shortly after initiation of DNA replication [96]. (**B**) Adaptation of (**A**) with the terminus-containing loop in the length axis of the cell. (**C**) Organization of DNA subregions before termination of DNA replication. Arrows indicate the direction of movement of DNA (towards the replisome or away from the replisome in the length axis of the cell). Black circles, *oriC* region; black squares, terminus region; yellow pentagons, *ftsQAZ* region; blue triangles, *minB* region; red dashed line, newly synthesized DNA. Note that, as replication progresses from (**B**) to (**C**), *minB*, moving towards the replisome, passes one of the *oriC*s, which is moving away from the replisome. Source: Modified Figure 5 of [88]. © John Wiley and Sons.

In Figure 19, fluorescent FISH spots are shown together with the DAPI-stained nucleoid. The distance of the center of a fluorescent focus to midcell was measured in a large number of cells. They were grouped according to length so that the position of the foci could be correlated with the known progression of DNA replication. In the interpretive scheme (Figure 20), all three gene regions (*oriC*, *ftsQAZ* and *minB*) are indicated as based on the model of Dingman (1974 [96]). That is, replisomes are located in a replicating apparatus or replication factory wherein DNA strands move apart after replication. In *E. coli* (Koppes et al. 1999 [97]), as well as in *B. subtilis* (Lemon and Grossmann, 1998 [98]), the replication center is in the cell center. The latter authors employed a GFP fusion with the catalytic subunit PolC of DNA polymerase.

In our case, we pulse-labeled cells with [^3^H] thymidine for 10 min before preparing for electron microscopic autoradiography. The resulting silver grains were scored in relation to cell length. It was found that incorporation of [^3^H] thymidine occurred predominantly in the cell center. This indicated that the replisomes are active at a fixed place and that they do not move along the chromosome. However, the model of Dingman with a fixed replication factory might be too simple. Recent considerations have been expressed by Woldringh (2023) [58].

What can be said about gene localization and its expression in the cell? Returning to Figure 20, it emerges that the cellular position of the cell division genes is largely determined by the DNA segregation status of the chromosome. However, this does not reveal when the gene cluster is expressed and to what extent. Moreover, FtsQ, A, and Z are likely produced in different quantities, in the 2-min region. With respect to the *FtsLBQ* cluster, *ftsB*, for instance, is located at 48 min. This also requires coordination regarding its synthesis. Presumably, gene regions are spatially positioned in the cell in an (unknown) dynamic way and their products are able to travel in the plane of the cell membrane, guided or not guided. Anyway, when the products have arrived in the cell membrane, they meet an existing organization. These are only a few thoughts that come to one’s mind. Obviously, what lies ahead is a tanglewood of regulations involving a plethora of promoters and concentrations of gene products.

## 13. Cell Elongation

### 13.1. Localization of PBP2

**Figure 21:**
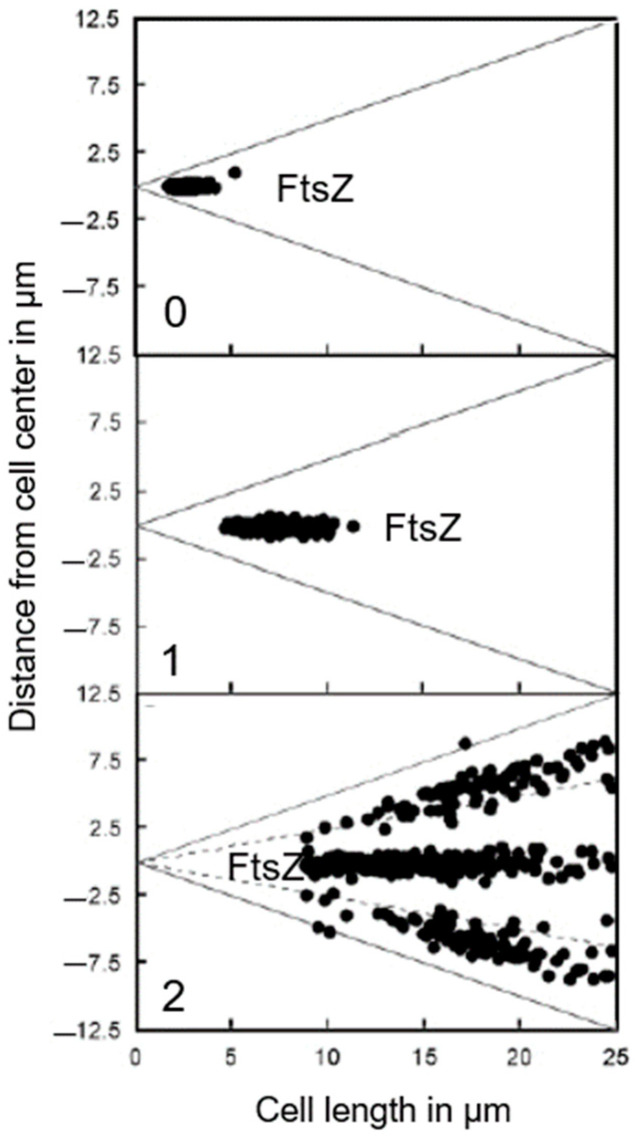
Inhibition of PBP3 by aztreonam. The FtsZ ring is not able to dissociate. The cells were grown to steady state in GB1 medium at 28 °C with a doubling time of 80 min and, subsequently, aztreonam was added (final concentration 1 µg/mL). The culture was sampled just before the addition of aztreonam (0 mass doubling), after one mass doubling (1) and after two mass doublings (2) of growth in the presence of aztreonam, fixed and immunolabelled with Alexa 546-conjugated Fab against FtsZ. The distance from the midcell position of the FtsZ rings is plotted as a function of cell length. Negative values refer to the arbitrary left side of the cell, and positive values refer to the arbitrary right side of the cell. The grey lines represent the border of the cells, and the dotted grey lines represent the one-quarter and three-quarter positions of the cells. Source: Modified Figure 5 of [99]. © John Wiley and Sons.

Where is PBP2 located in the cell? Localization studies of a GFP-construct of PBP2 led to some surprising results (den Blaauwen et al., 2003 [99]). The PBP2 label was clearly observed at the cell center (not necessarily in a divisome), apart from lateral location (elongasome), as expected. The central label of GFP-PBP2 disappeared upon inhibition of PBP3 with aztreonam, whereas FtsZ remained present (Figure 21). As pointed out before: “This suggests that PBP2 localization at the divisome is dependent on active PBP3” (den Blaauwen et al., 2003 [99]). Furthermore, GFP-PBP2 seemed not to be part of PIPS (see below) in the presence of aztreonam. Nevertheless, what does the central location of GFP-PBP2 mean? I will return to this point later on, after having referred to the association of PBP2 with the actin-like MreB.

### 13.2. Cell Elongation Participants

Actin-like cytoplasmic helical filamentous structures underneath the lateral envelope of MreB in *B. subtilis* (Jones et al., 2001 [100]), and later in *E. coli* (Shi et al., 2003 [101]), revealed the existence of a cytoskeletal element next to the tubulin-like FtsZ. The gene *mreB* and its neighbours *mreC* and *mreD* had already been found in 1987 (Wachi et al., 1987 [102]). Together they form a membrane-bound complex, whereby MreB interacts with MreC, and MreC with MreD (Kruse et al., 2005 [103]). Briefly, they play a role in shape maintenance, together with other components, notably PBP2. PBP2 also interacts with RodA (Stoker et al., 1983 [104]), which is a transglycosylase (Rohs et al., 2018 [105] and references therein).

MreB rotates around the circumference of the cell in coordination with the assembly of peptidoglycan (van Teeffelen et al., 2011 [106]), thus representing the (dynamic) elongasome counterpart of the divisome with FtsZ and PBP3. Substrates (lipid II) for PBP2 are prepared by MurG, a peptidoglycan glycosyltransferase, as mentioned before (Figure 11). MurG occurred all over the envelope, with some accent at the site of division after immunofluorescence microscopy, and it appeared in a complex with MreB and MraY (Mohammadi et al., 2007 [107]). According to the authors, the localized supply of lipid II, as provided by MurG, has to be at the elongasome as well as the divisome. With respect to the divisome, the localization appeared to be PBP3-dependent. This suggests, but does not prove, that peptidoglycan synthesis before PBP3 becomes active, and uses MurG in an MreB environment in the cell center.

**Figure 22:**
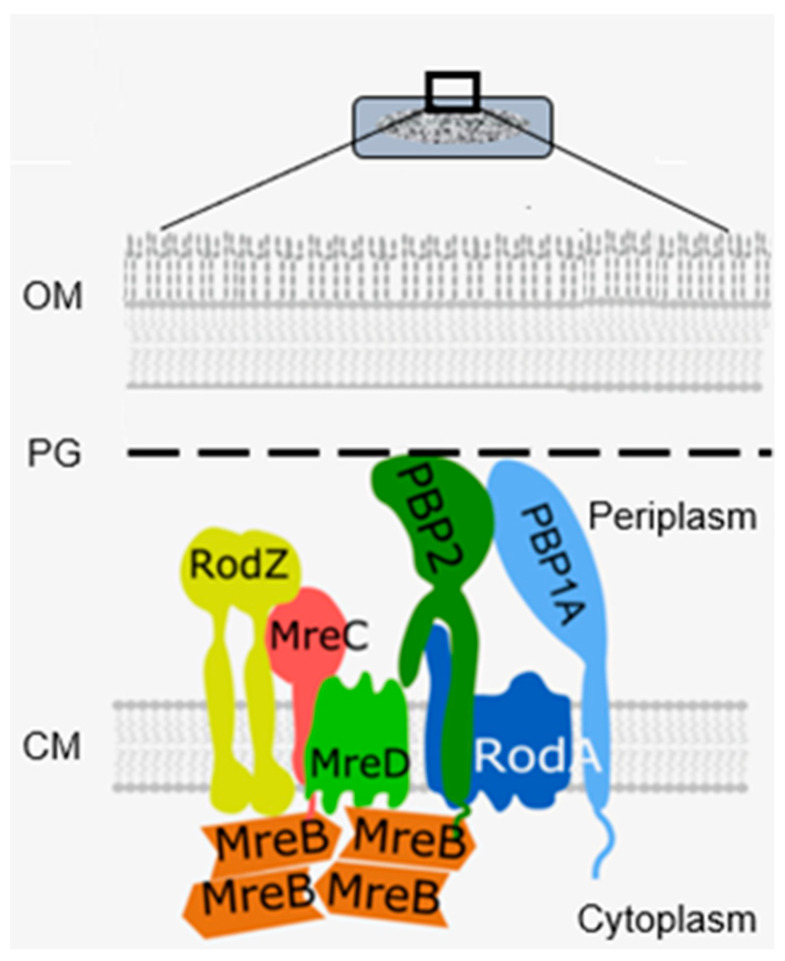
Core elongasome proteins in *E. coli*. Proteins are recruited by MreB. CM, cell membrane; OM, outer membrane; PG, peptidoglycan. Source: Modified Figure 1 of [109]. Open access.

Another problem regards the nature of the flippase that translocates lipid II to the periplasm. Confusion has arisen as to whether FtsW or MurJ acts as such. It now appears that FtsW functions as a flippase in vitro (Mohammadi et al., 2014 [108]), whereas this applies for MurJ in vivo (Liu et al., 2018 [109]). MurJ has a location similar to that of MurG. To become located at the divisome, it requires lipid II synthesis and active FtsW; to be located at the elongasome, it possibly requires RodA instead of FtsW (Liu et al., 2018 [109]). Another protein, RodZ, is also involved in shape geometry of *E. coli* (Shiomi et al., 2008 [110]). Its precise function has still to be elucidated. Figure 22 shows a recent version of an elongasome model.

## 14. PIPS

**Figure 23:**
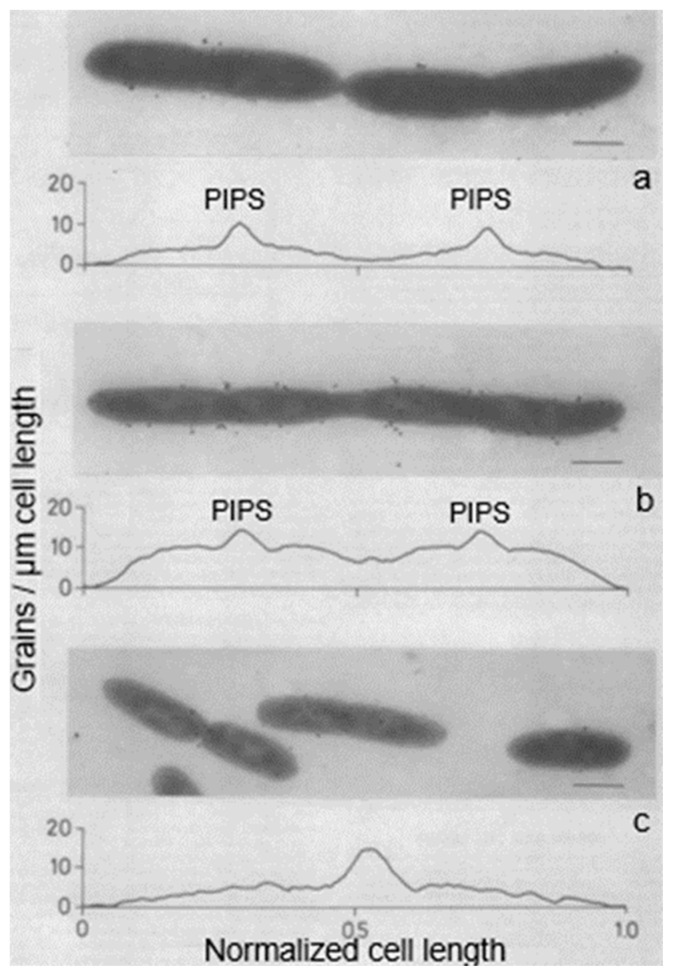
Topography of [^3^H] Dap incorporation after inhibition of PBP3 and PIPS. (**a**) The temperature-sensitive cell division mutant MC4100 *lysA pbpB* was grown at 28 °C in the presence of 2µg of furazlocillin per mL. Grain distribution of furazlocillin-induced filaments with three constrictions (*N* = 130; G = 4876; *L* = 10.68 µm, where *N* is the number of cells, *G* is the number of grains, and *L* = 12.16. (**b**) Cells grown at the restrictive temperature at 37 °C without furazlocillin. Grain distribution of temperature-induced filaments with three constrictions (*N* = 164; *G* = 15,506; *L* = 12.16 µm). (**c**) Grain distribution of normally dividing *pbpB* cells at the permissive temperature (*N* = 92; *G* = 1533; *L* = 4.11 µm). Bar, 1 µm. Source: Modified Figure 6 of [57]. Copyright ©1989, American Society for Microbiology.

Inhibition of PBP3 by mutation (a temperature-sensitive *pbpB* mutation) or by furazlocillin, aztreonam, or cephalexin (specific ß-lactam antibiotics for PBP3) produced filaments with blunt constrictions. Upon further growth, the central blunt constriction became flanked by two new normal-looking constrictions (Figure 23; Wientjes and Nanninga, 1989 [57]). I quote: “Inhibition of PBP2 (by mecillinam) and PBPs 1A and 1B (by cefsoludin or moenomycin) does not prevent initiation of division (our unpublished observations). These observations point to the involvement of a penicillin-insensitive peptidoglycan-synthesizing activity (PIPS) during initiation of constriction“ (Nanninga, 1991 [111]). I have to make one correction here: “our unpublished observations” are now “my lost observations”. I would like to emphasize here that PIPS refers to constricting cells. It appears possible that PIPS still interacts with FtsZ upon inhibition of PBP3, because constrictions are well visible (Figure 21 and Figure 23 and see below). Temperature-sensitive FtsZ filaments are smooth at the non-permissive temperature and no localized peptidoglycan synthesis takes place in that case, as deduced from autoradiograms (Woldringh et al., 1987 [56]).

With a novel technology, filamentous *E. coli* sacculi obtained from cells with inhibited PBP3 also showed three division sites (a central one and two flanking ones). In this case, cells were grown in the presence of D-cysteine so that sacculi containing modified peptidoglycan with -SH groups could be biotinylated. The latter were detected in the electron microscope by antibiotin antibodies conjugated to gold particles. The two flanking division sites mentioned above, with active peptidoglycan synthesis, became visible by the absence of label after an extensive chase (de Pedro et al., 1997 [112]), thus confirming the presence of PBP3-independent peptidoglycan synthesis at division sites. See, however, below.

## 15. PIPS and Pre-Septal Peptidoglycan Synthesis (PPS)

### 15.1. Definition of PIPS

In a recent paper, the following description of PIPS and PPS can be found: “For the purpose of this study, we refer to PIPS as pre-septal PG synthesis” (Pazos et al., 2018 [113]). Also, in an earlier paper, no distinction was made between PIPS and PPS (Potluri et al., 2012 [114]). Pre-septal PG synthesis I have denoted as PPS, for short. I am afraid that combining PIPS and PPS might be confusing. Briefly, the distinction between the two is based on the presence or absence of a constriction, respectively. Below, I will try to elucidate the distinction.

As mentioned before, PIPS arose after inhibition of PBP3. Strictly, it should be defined as PBP3-inhibited peptidoglycan synthesis. Note that [^3^H] Dap is incorporated, as can be seen in the autoradiogram (Figure 23). Thus, what kind of peptidoglycan synthesis is applicable? Can one maintain that, say, PBP2, is not involved? This appears a valid question because the elongasome has to be replaced by a (nascent) divisome at the cell center. Since PIPS does not require PBP3, will PBP2 carry out the job? Below, this will be adressed further.

### 15.2. Pre-Septal Peptidoglycan Synthesis (PPS)

**Figure 24:**
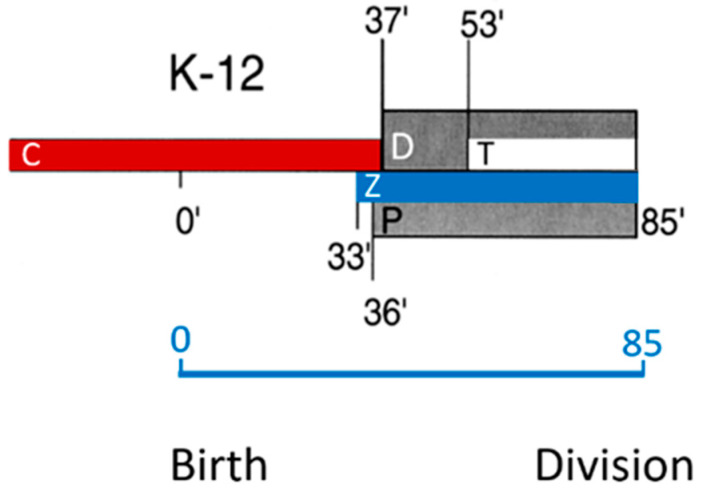
Schematic representation of the cell cycle of *E. coli* K-12 growing with a generation time of 85 min. The C period shows the duration of the DNA replication cycle. Note that C starts before birth. Thus, the cell cycle starts in the middle of C. The D period is the time between the termination of DNA replication and the separation into two daughter cells. The Z period shows the duration of the period in which FtsZ can be detected at midcell. The P period shows the period of peptidoglycan synthesis during constriction [116], and the T period is the duration of the constriction. Source: Modified Figure 7 of [115]. Copyright © 1989, American Society for Microbiology.

I now turn to pre-septal peptide glycan synthesis (PPS). “Pre-septal” means before division (of course), implying that, as soon as a constriction emerges, the term pre-septal does not apply anymore. What unites the activities of PPS and PIPS is that they both require FtsZ, are located at the cell center, and occur before PBP3 comes to the fore (den Blaauwen et al., 2003 [99]). To further delineate PPS and PIPS, it will be helpful to look at their temporal position in the cell cycle (Figure 24; den Blaauwen et al., 1999 [115]). This refers to a steady-state culture of *E. coli* K-12 MC4100 with a doubling time of 85 min at 28 °C. These conditions have been used by us again and again to make experiments comparable which were carried out in the course of time.

Note that, in Figure 24, DNA replication (C-period) starts in the previous cell cycle and finishes at 37 min in the ongoing one. Thus, a newborn cell contains a half-replicated chromosome. The D-period lasts from termination of DNA replication to cell separation, and its duration is thus 85 minus 37 min equaling 48 min. The T-period represents the visible duration of cell division (85 minus 53 min equals 32 min). FtsZ (Z-period) becomes visible at 33 min, i.e., around termination of DNA replication and the start of peptidoglycan synthesis at the cell center (P-period). The duration of the P-period has been taken from Taschner et al. (1988 [116]).

How do PIPS and PPS fit into this scheme? Since PIPS refers to constricting cells, it is located at the beginning of the T-period (the remainder of the T-period refers to PBP3-dependent peptidoglycan synthesis). What about PPS? Visibility of constriction (beginning of the T-period) starts at 53 min, so this is the end of PPS. PPS starts with the beginning of the P-period. The latter more or less coincides with the end of DNA replication (beginning of D) and central positioning of FtsZ (Aarsman et al., 2005 [117]; Pazos et al., 2018 [113]). FtsZ is present during PPS and PIPS.

### 15.3. A Closer Look at Autoradiography

With this in mind, we can have another look at Figure 14. It depicts silver grain distributions measured in autoradiograms of *E. coli* K-12 cells pulse-labeled with [^3^H] Dap. Thus, the uninterrupted line represents peptidoglycan synthesis in T-period cells and the interrupted line includes all non-dividing cells. Likely, the central peak in the latter case reflects PPS. We can also have another look at immuno-labeled sacculi mentioned above. My interpretation is that they reflect a combination of PPS and PIPS. There are two arguments for this interpretation. Firstly, PIPS is about pulse-labeling in a local area, that is, what we have termed the leading edge of constriction (Wientjes and Nanninga, 1989 [57]). Secondly, the less-labeled zones visible after immunolabeling (de Pedro et al., 1997 [112]) are rather broad, as compared to a leading edge.

In summary, for the time being, we might distinguish three stages during cell division: PPS, PIPS, and a divisome core activity (Kashämmer et al., 2023 [118]), of which PBP3 is a main participant. Below, I will attempt to further delineate participants of PPS and PIPS.

### 15.4. Participants of PPS

PPS starts in cells where a functional transition can be expected at the cell center from elongasome to divisome. At the cell center, the bacterial actin filaments of MreB and the tubulin-like filaments of the GTPase FtsZ appear to interact directly (Fenton and Gerdes, 2013 [119]), and the latter authors suggested that MreB bound to FtsZ still stimulated PBP2 and PBP1B activities to produce peptidoglycan. However, other authors stressed the interaction between PBP2 and PBP1A (Banzhaf et al., 2012 [120]) and the interaction between PBP3 and PBP1B (Wientjes and Nanninga, 1991 [75]; Bertsche et al., 2006 [121]; Ranjit et al., 2017 [122]; Boes et al., 2019 [123]). One might speculate that upon the earliest arrival of FtsZ to the cell center, MreB-directed peptidoglycan synthesis, aided by PBP2 and PBP1A and/or PBP1B, synthesizes PPS, at least in part.

### 15.5. Participants of PPS and PIPS

Earlier work (Figure 24) has shown that FtsZ is positioned at the cell center about 20 min before a constriction becomes visible in an *E. coli* K-12 LMC500 (MC4100*lysA*) strain, thus during PPS. Attachment of FtsZ to the cell membrane is facilitated by FtsA (Ma et al., 1996 [124], 1997 [125]; Wang et al., 1997 [126]; Pichoff and Lutkenhaus, 2002 [127]) and by ZipA (Hale and de Boer, 1997 [128]; Pichoff and Lutkenhaus, 2002 [127]; Potluri et al., 2012 [114]) implying that they are the first components of the nascent or proto-divisome.

According to Potluri et al. (2012) [114], ZipA is required for PPS/PIPS peptidoglycan synthesis. Note that I am using here the term PPS/PIPS as I have argued above. In their comprehensive study, they also investigated other possibilities for peptidoglycan synthesis in the absence of PBP3 and without involving PBP2. They took into account the glycosyltransferases PBP1C (Schiffer and Höltje, 1999 [129]), a peptidoglycan polymerase that catalyzes glycan chain elongation from the lipid-linked precursor MtgA (Di Berardino et al., 1996 [130]) and the penicillin-insensitive L,D-transpeptidases YnhG and YcbB (Magnet et al., 2007 [131], 2008 [132]; Höltje, 1998 [47]; Vollmer and Bertsche, 2008 [133]) and they made a strain with all four mutations. It appeared that so-called PIPS bands were still formed. In addition, not required for PPS/PIPS are AmiC (N-acetylmuramyl-L-alanine amidase), EnvC (murein hydrolase activator), and seven ß-lactamases (PBP1A, PBP4, PBP5, PBP6, PBP7, AmpC (Class C ß-lactamase), and AmpH (Class C ß-lactamase)) in a mutant lacking all of them [114].

Thus, after FtsZ, ZipA, FtsA, and PBP1A and/or PBP1B have taken over, a first step in divisome assembly has been completed (Pazos et al., 2018 [113] and references therein). The role of PBP2 has still to be clarified further (see below).

### 15.6. Participants of PIPS

**Figure 25:**
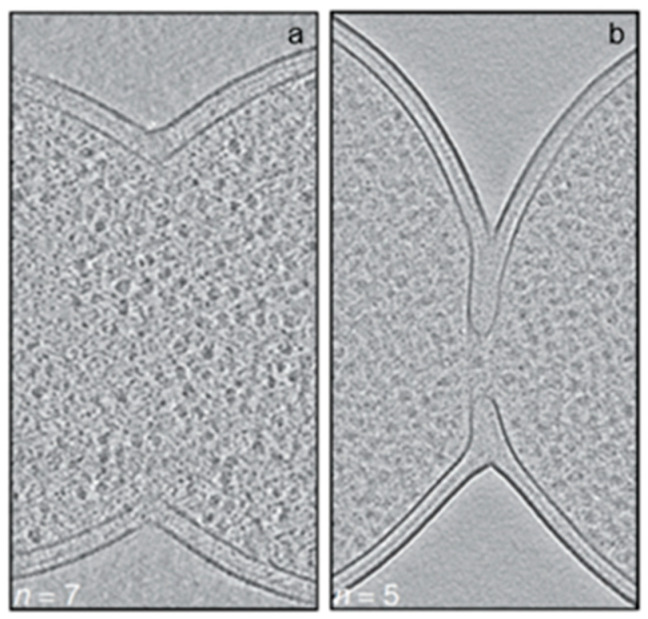
Two different stages of division in wild-type *E. coli*. (**a**) Initiated division. The envelope layers are still together. (**b**) Progression of division with envelope layers disconnected. Summed, projected central slices of cryo-electron tomograms. Source: Figure 1 (partly) of [134]. Open access.

With PIPS, the constriction becomes visible in the electron microscope. What does the constriction look like? For this purpose, we can examine recent cryo-sections for the electron microsope (Navarro et al., 2022 [134]). What can be observed is the tight organization of the envelope layers during initial constriction (Figure 25a). Presumably, this is also the case with PIPS. Figure 25 has been selected to address the point I am making here. For numerous other interesting details, the reader should consult the original paper (Navarro et al., 2002 [134]).

As implied above, PPS is probably facilitated by PBP2 as long as there is no constriction. Nevertheless, does PBP2 have a role for PIPS while PIPS constrictions still occur after inhibition by mecillinam? My speculative answer is based on the experiments of Wientjes and Nanninga (1991) [75], where it was shown that the inhibition by mecillinam was not complete, that is, around 65 percent. In other words, is the remaining 35 percent activity of PBP2 still able to play a role in the initiation of constriction? I will return to this point after having discussed the assembly of the remaining divisomal proteins.

Returning to Figure 25b, it seems that the outer membrane is lagging behind, whereas the peptidoglycan layer is less distinct. Interaction of lipoproteins with the outer membrane seems not very possible here. In particular, would this apply to LpoB (see below) and its partner PBP1B? However, the question can be posed whether, in this stage, PBP3 is still there. Perhaps this is not the case, and are preparations made to separate the daughter cells in the absence of FtsZ (Söderström et al., 2014 [135])?

## 16. Remaining Divisomal Proteins

**Figure 26:**
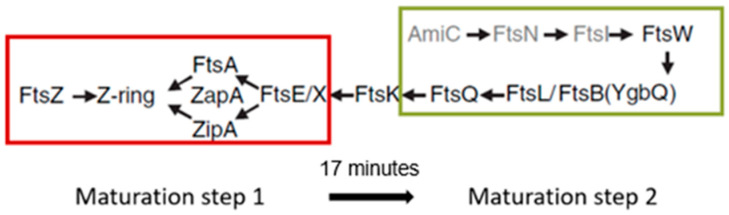
Two maturation steps of divisome assembly. Adapted from [86,117].

So far, I have dealt with the early preparations of the division process, i.e., the assembly of the nascent divisome. How does the composition of the nascent divisome change during the course of preparation and execution of the division process? After FtsA and ZipA, additional proteins become incorporated into the nascent divisome. The sequence is known, as well as the interdependency regarding their interaction (Figure 26; Buddelmeijer and Beckwith, 2002 [86]; Aarsman et al., 2005 [117]). However, this sequence is to some extent misleading because it suggests that most proteins follow one after another while constituting the divisome. In fact, some proteins are grouped independently before they enter the divisome. This applies, for instance, to a trimeric complex of FtsL, FtsB and FtsQ (Buddelmeijer and Beckwith, 2004 [136]).

So, when do the remaining divisomal proteins arrive at their final destination? To answer this question, the fraction of fluorescently labeled cells at the end of the cell cycle was determined. In this way, the timing of the arrival of divisomal proteins could be calculated from their labeled fractions (Aarsman et al., 2005 [117]). This has been done for FtsQ, FtsW, PBP3, and FtsN. It appeared that, under the growth conditions used (doubling time 85 min at 28 °C), all of them arrived at about the same time at the divisome, i.e., about 17 min after the first positioning of FtsZ. Thus, the authors proposed two maturation steps for divisome assembly (Figure 26).

Since PIPS precedes PBP3 activity, by definition, the continuation of initial constriction is likely based on peptidoglycan synthesis of the PBP3 (transpeptidase)-FtsW (transglycosylase; Taguchi et al., 2019 [137]) couple. A complex of PBP3-FtsW had already been found independent of other divisomal proteins (Fraipont et al., 2011 [138]). Conceptually, this complex resembles that of PBP2 and RodA of the *E. coli* elongasome (Ishino et al., 1986 [139]; Rohs et al., 2018 [105]). Returning again to Figure 25b, one wonders whether the outer membrane is still at its original location, that is, tightly bound to the peptidoglycan layer. Anyway, FtsZ leaves the Z-ring before the end of cell separation, while FtsA, ZipA, FtsQ, FtsL, and PBP3 are still present (Söderström et al., 2014 [135]).

## 17. Constructing the Core Divisome

Recently, a pentameric complex (FtsWIQBL) composed of the trimeric FtsL, FtsB, and FtsQ and the PBP2-FtsW couple, both from *Pseudomonas aeruginosa*, was purified and frozen. Subsequently, its structure was determined by cryo-electron microscopy at a resolution of 3.7 Ä (Kashämmer et al., 2023 [118]). It will be observed that FtsK and FtsN are not yet incorporated. Presumably, this will be a matter of time. As pointed out by the authors: “As FtsWIQBL is central to the divisome, our structure is foundational for the design of future experiments elucidating the precise mechanism of bacterial cell division, an important antibiotic target”.

## 18. A Role for Outer Membrane Proteins LpoA and LpoB

Though peptidoglycan metabolism is generally viewed in the direction from cytoplasm to cell membrane, and from there to the peptidoglycan layer (Figure 11), recent research introduced the direction from outer membrane to peptidoglycan layer and cell membrane (Typas et al., 2010 [140]). This was based on the characterization of two new lipoproteins, called LpoA and LpoB. Briefly, they interact with PBP1A and PBP1B, respectively, to bind new peptidoglycan to the sacculu*s.* Thus, LpoA and LpoB presumably have mainly to do with the elongasome and the divisome, where they interact with the transpeptidase moieties of the respective PBPs1.

## 19. PPS and PIPS (Preparative Cell Division) and the Core Divisome

Under this heading, I will attempt to integrate the above constituents of the division process. This will be mainly based on the paper of van der Ploeg et al. (2013) [141], entitled “Colocalization and interaction between elongasome and divisome during a preparative cell division phase in *Echerichia coli*”. It is, amongst others, an advanced continuation of a paper which appeared one year after my tenure in 2002 (den Blaauwen et al., 2003 [99]). I also wish here to acknowledge the excellence of Tanneke den Blaauwen while pursuing this topic with the persons she assembled around her. I am proud she has continued the philosophy of the Amsterdam School mentioned before.

**Figure 27:**
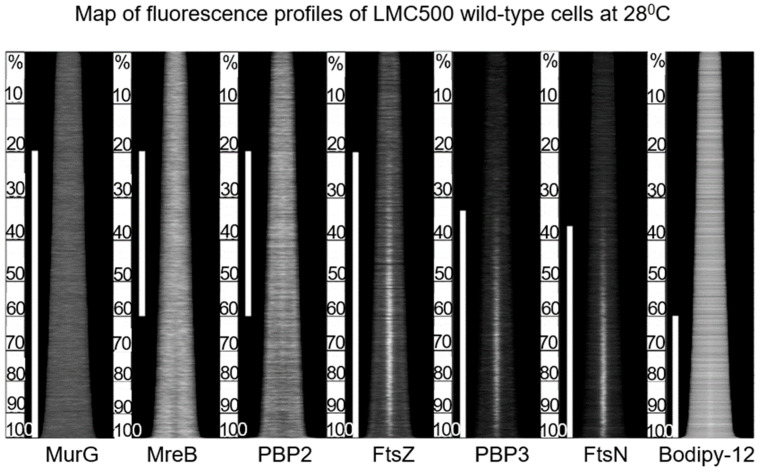
Maps of fluorescent profiles of immunolocalized endogenous elongasome and divisome proteins MurG, MreB, PBP2, FtsZ, PBP3, and FtsN of *E. coli* LMC500 wild-type cells grown to steady state in GB1 medium at 28 °C. The integral fluorescence of each cell is plotted as a function of cell length (*x*-axes) and all cells are plotted with increasing age (*y*-axes). The white bars present midcell localization judged by visual inspection. Bodipy-12 is a general membrane stain. Source: Modified Figure 2 of [141]. © John Wiley and Sons.

**Figure 28:**
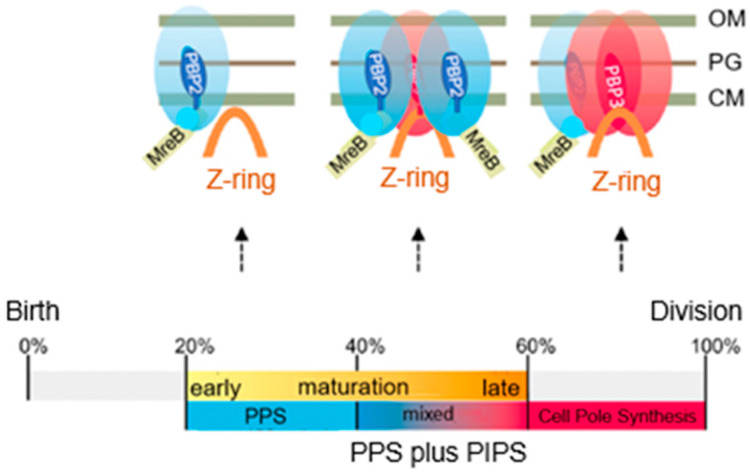
Transition from elongasome (PBP2) to divisome (PBP3) activity at midcell. Growth conditions as in Figure 27. FtsZ is present all the time. During the transition PBP2 and PBP3 are both located at midcell in the presence of MreB and FtsZ. At the onset of cell pole synthesis, PBP2 disappears from the cell center. By contrast, pre-septal peptidoglycan synthesis (PPS) is dependent on PBP2. In between, a mixed situation arises, which I interpret as PPS plus PIPS. PIPS also requires a transglycosylase activity which could be provided by RodA and/or PBP1A. The time scale is divided in percentages to indicate the events occurring from birth to division. CM, cell membrane; OM, outer membrane; PG, peptidoglycan layer. Source: Modified and based on Figure 6 of [141]. © John Wiley and Sons.

The above study involved the immunolocalization of MurG, MreB, PBP2, FtsZ, PBP3, and FtsN during the cell cycle (maps of fluorescent profiles) as well as Förster Resonance Energy Transfer (FRET) of PBP2 and PBP3. Remember that MurG catalyzes the transfer of lipid I to lipid II (Figure 11). The maps of fluorescent profiles can be seen in Figure 27 of endogenous elongasome and divisome proteins. A vertical white bar indicates (visually) the central position of a label. Note, that MreB and PBP2 localize more or less together in the cell center, and that FtsZ starts at about the same time (cell cycle fraction) as PBP2. PBP3 enters much later in the midcell and FtsN follows. Also note that the positions of PBP2 and PBP3 overlap, which might explain the above-mentioned FRET data. Their data have been summarized in Figure 28.

The model addresses aspects related to the enigmatic PIPS. To summarize: the FtsZ ring is there all the time, as is MurG. MreB starts to disappear, as does PBP2. The latter is still there when PBP3 becomes involved in cell pole synthesis after the late maturation step of the divisome assembly. We also find PPS commencing the early phase of divisome assembly. After PPS follows a mixed zone which includes PIPS and PBP2, a transpeptidase. Presumably, RodA provides for transglycosylase activity, enabling peptidoglycan synthesis after inhibition of PBP3 with aztreonam. In other words, it is tempting to speculate that PIPS is constituted of PBP2 and RodA.

## 20. Final Steps

There is a structural continuity between nucleoid and envelope, as mediated by coupled transcription, translation, and insertion of membrane proteins in the cell membrane. Eventually, DNA catenanes in the cell center have to be broken after termination of DNA replication to allow the new nucleoids to find their positions in the daughters. Clearly, DNA should be occluded from the cell center for division to be completed (Mulder and Woldringh, 1989 [142]); hence the later term nucleoid occlusion (NO). One way to occlude fission through the nucleoid would be a division inhibitor that prevents Z-ring assembly “on portions of the membrane surrounding the nucleoid” (Bernhardt and de Boer, 2005 [143]). They found a DNA-associated division inhibitor denoted as SlmA. Another negative regulation is carried out by the Min system (MinC, MinD, and MinE), which prevents the formation of minicells at the poles (Lutkenhaus 2007 [144] and references therein).

A positive regulation mechanism has to do with the DNA-binding protein MatP, which organizes the replication terminus region, the Ter linkage (review: Männik and Bailey, 2014 [145]). MatP can bind to Zap B, which in turn binds to ZapA located on FtsZ (see later). Finally, FtsK, when present in the divisome, can translocate DNA with the aid of ATP when it is in a hexameric assembly (Sheratt et al., 2010 [146]). Presumably, such translocations change the higher-order structure of the nucleoid in such a way that its segregation at the end of DNA replication is facilitated.

The above systems linking DNA segregation to cell division are largely present in cells growing in a rich medium as contrasted to slowly growing cells (Männik and Bailey, 2014 [145]). It would seem that fast-growing cells (presumably, multifork DNA replication) require more coordination to achieve safe cell proliferation. Obviously, according to the authors, the last word has not yet been said on this topic: “Our data, however, is indicative that yet an unidentified, lower fidelity positioning system remains in *E. coli* ΔslmA Δmin cells even without the Ter linkage” [145].

## 21. What Is the Mechanical Role of FtsZ Polymers during Constriction?

### 21.1. Role of FtsZ

In the foregoing, I have focused on peptidoglycan assembly and its role in the cell division process. However, what can FtsZ do by itself? Constriction starts with bending of the cell membrane in the presence of the peptidoglycan layer and the outer membrane (Figure 25a). So, what causes membrane bending? Is there a pulling force based on FtsZ as a participant? Next, the constriction proceeds while building two hemispherical domes from the bended surfaces. Is there a shape-maintaining skeleton? Finally, is there a force that results in cell separation? I will start with two opposing models and then try return to the questions formulated above.

**Figure 29:**
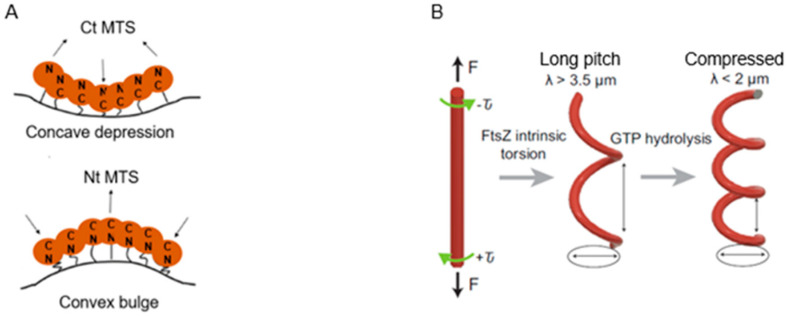
Autonomous bending of FtsZ protofilaments. (**A**) A model of membrane deformations generated by bending force of FtsZ filaments. When the membrane-targeting sequence (mts) is attached at the C- or N-terminus, the bent protofilaments form a concave depression (left panel) or convex bulge (right panel), respectively. The direction of bending to make a concave depression is the same as that of Z-ring constriction. Source: Modified Figure 6B of [149]. (**B**) An elastic rod subjected to a constant force (F) reveals a helical deformation upon twisting. Intrinsic FtsZ torsion rules long-pitch transformations (λ  >  3 µm) while GTP enhances further torsion, causing higher pitch states (λ  <  2 µm). Source: Modified Figure 3f of [150]. (**A**). © John Wiley and Sons.; (**B**) Open access.

Model one starts from the basic observation that FtsZ protofilaments can form upon GTP binding (Mukherjee and Lutkenhaus, 1994 [147]) and that the protofilaments are attached to the cell membrane. Membrane anchors are FtsA and ZipA in the living *E. coli* cell. The C terminus of FtsZ binds FtsA, and the amphipathic helix at the C terminus of FtsA represents its membrane anchor (cf. Osawa et al., 2008 [148]). In a model system, Osawa et al. (2009) [149] used liposomes and, instead of FtsA, a membrane-targeted FtsZ construct called Fts-mts. Inside the liposome tubules, Fts-mts could form constricting Z rings, indicating that the Z rings can generate a force independently of other proteins. Later studies showed that a concave or convex membrane impression could be obtained dependent on whether the mts side was at the C terminus (the normal side) or at the N terminus, respectively (Osawa et al., 2009 [149]; Figure 29A). These in vitro experiments suggest that FtsZ protofilaments can bend liposomal membranes.

A somewhat different approach was followed by Ramirez-Diaz et al. (2021) [150]. They pulled so-called soft lipid tubes from giant lipid vesicles with optical tweezers. The giant lipid vesicles had been decorated with an FtsZ-mts strain containing a yellow fluorescent protein (YFP) as a label. Remarkably, conformational alterations of the tubes’ surfaces occurred by torsional stress after GTPase activity. It has been speculated that torsional stress in FtsZ protofilaments might also contribute to constriction of *E. coli* cells (cf. Figure 29B).

### 21.2. Role of Peptidoglycan

Model two switches from FtsZ to peptidoglycan as an organizer of constriction (Coltharp et al., 2016 [151]). They showed that the rate of constriction was reduced by a mutation of PBP3 and not so much as by a mutation of FtsZ. The results were interpreted to mean that peptidoglycan remodelling by PBP3 is instrumental for the constriction force. Thus, in contradiction to the above models, the constriction has been supposed to arise from the outside (PBP3) and not from the inside (FtsZ) of the cell.

It appears relevant that, in the first case, we are looking at in vitro models where one always has to find out whether they apply to an in vivo situation. By contrast, the second model is based on presumed peptidoglycan synthesis, though measured in a very indirect (non-biochemical) way. However, it has already been known for a very long time that inhibition of PBP3 leads to impairment of the division process, presumably because PBP3 is a component of the multilayered divisome-encompassing cytoplasm, cell membrane, peptidoglycan layer, and outer membrane. Making peptidoglycan synthesis responsible for constriction seems too simple a conclusion, in particular in light of what is already known about FtsZ protofilaments. It should also be noted that the authors were primarily focused on measuring completion of constriction, and not its initiation (PSS/PIPS). The PSS/PIPS system operates, as I have indicated, prior to the functioning of the core divisome, which includes PBP3 in the presence of FtsZ. The also-present FtsW (transglycosylase) might help in maintaining the shape of the nascent hemispherical domes through interaction with FtsZ. Presumably, the domes are reinforced by nascent peptidoglycan, though the latter might disappear upon last stages of cell separation (Söderström et al., 2014 [135]). Cell separation might be an enzymatic process without the requirement of mechanical assistance.

### 21.3. Role of Phospholipids

The above dealt with bending of the cell membrane with various external agents. But, what about the cell membranes themselves? For instance, their phospholipid composition, that is, are the hemispherical caps that arise during constriction different from the lateral cell membranes? As an approach, we isolated minicells, assuming that they are constructed of two hemispherical caps (an old one and a new one). *E. coli* LMC500*lysA* was used as the wild-type strain, and *E. coli* LMC1088 was used as a minicell-forming mutant. Cells were grown to steady state with a doubling time of 80 min at 28 °C in glucose minimal medium (our standard conditions). Minicells were separated by centrifugation from LMC1088. Phosphatidylethanolamine (PE), phosphatidylglycerol (PG), and cardiolipin (CL) concentrations were determined in the wild type, in LMC1088, and in minicells. PE was a dominant species in all samples, whereas the overall composition of the wildtype and LMC1088 was very similar. However, minicells contained more Cl and less PG (Koppelman et al., 2001 [152]). These results suggest that cardiolipin might aid in membrane bending. At the moment, this is still a speculation.

## 22. Divisome Subassemblies

### 22.1. Subassemblies

**Figure 30:**
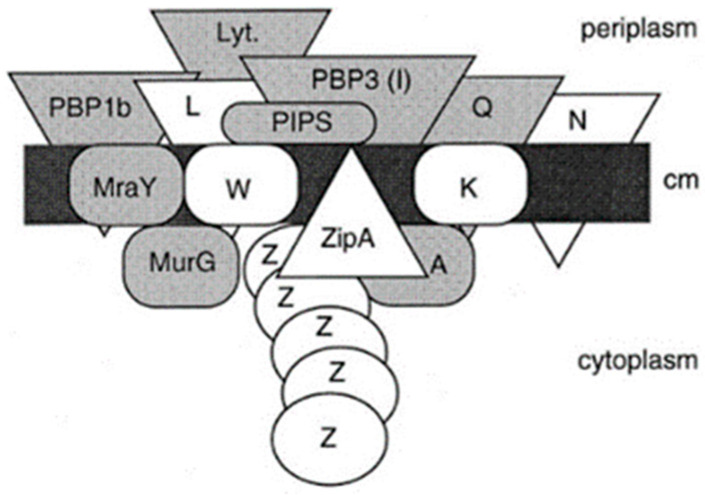
Model of divisome subassembly. It unites cytoplasm and periplasm. The gene products FtsA, FtsI (PBP3), FtsK, FtsL, FtsN, FtsQ, FtsW, and FtsZ have been denoted as A, I, K, L, N, Q, W, and Z, respectively. Lyt. indicates that lytic enzymes must be present; cm, cell membrane. Source: Figure 11 of [44]. Copyright © American Society for Microbiology.

In the foregoing, divisome proteins have been treated individually or as interacting individuals. However, a higher order of organization should also be considered. For instance, how many pentameric complexes (FtsWIQBL; Käshammer et al., 2023 [118]) participate in the division process? How are their activities coordinated? Do their numbers change during division (Wientjes and Nanninga, 1989 [57])? As outlined before, most divisomal proteins occur in a limited number of copies per cell as compared with FtsZ, “implying that the FtsZ ring is not saturated with cell division proteins. Therefore, as a tentative model, one can envision that the divisome is composed of subassemblies which are connected by FtsZ polymers” (Figure 30; Nanninga, 1998 [44]).

Though the term subassembly might appear appropriate, they are certainly not connected by a continuous FtsZ polymer. The basic architecture of the subassembly encompasses components of the cytoplasm, cell membrane, and periplasm (including the peptidoglycan layer). In particular, components should be present, or nearby, that, as mentioned before, can perform rearrangements, turnover, recycling, degradation, and autolysis of peptidoglycan. Also note the presence of MraY (synthesis of lipid I), MurG (synthesis of lipid II) and a general lytic activity (Lyt.). The identity of the expected flippases (Figure 11) was then not yet known. Last but not least, PIPS is also included as a periplasmic enzymatic activity.

### 22.2. Stoichiometry of Subassembly Components

The above approach was based on the relative excess number of FtsZ molecules, that is, not all divisome proteins could cover FtsZ. To substantiate this further, more correct information is required about the concentration of divisomal proteins in the cell and in the divisome. Here, I mention two approaches. Earlier in this article, I stressed the importance of measuring cells according to various parameters, as developed by Norbert Vischer. I referred to the program ObjectJ. This was decades ago, and it was also used for decades. Now this expertise has been augmented by a specialized software project, Coli-Inspector, which is used to make fluorescent profile maps of cells arranged according to length and thus to age (Vischer et al., 2015 [38]). It applies to widefield microscopy and immunolabeling of wild-type K-12 strain MC4100 grown to steady state in minimal glucose medium at 28 °C. As stated before, these were our standard growth conditions which we maintained over the course of years.

**Figure 31:**
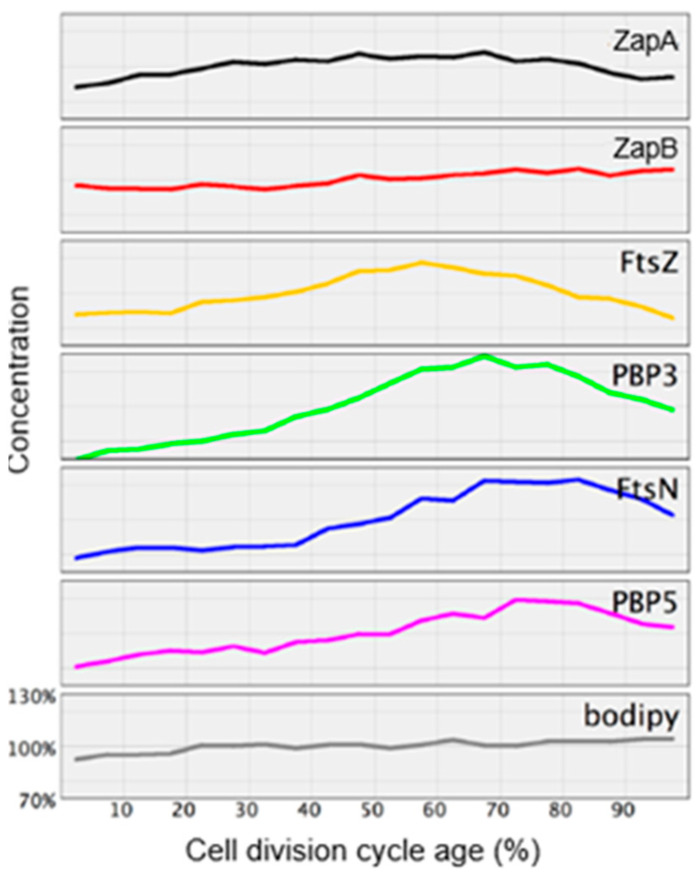
Protein concentration (normalized) as a function of cell age. It applies to ZapA, ZapB, FtsZ, PBP3, FtsN, and PBP5. Bodipy-C12 has been used as a general membrane stain. The concentration of each indicated protein was divided by the average concentration of that protein in the whole population. The individual proteins were plotted against cell division cycle age (%). Source: Figure 6 of [38]. Open access.

The amount of fluorescence per cell could be correlated with the concentration of a number of divisome proteins during the cell cycle. Briefly, knowing the number of proteins per cell (Li et al., 2014 [153]) allowed the calibration of fluorescence to the number of proteins per cell (and also at midcell). It can be seen in Figure 31 that the concentrations of ZapA and ZapB remain more or less constant during the division cycle. For FtsZ, PBP3, FtsN, and PBP5, roughly a maximum around 60, 70, 75, and 75 per cent can be observed, respectively, presumably mimicking their developing activities. Note that the authors did not find a concentration change for PBP3 and PBP5, as observed in the past (Figure 15; Wientjes et al., 1983 [74]). Since FtsZ peaks before PBP3, which makes sense, the present data are probably more trustworthy (after forty years).

Also based on the data of Li et al. (2014) [153], Egan and Vollmer (2015) [154] attempted to determine the stoichiometry of the core divisome proteins (including PBP3). The finding “that most proteins which participate in known multiprotein complexes are synthesized proportional to their stoichiometry” (Li et al., 2014 [153]) led them to derive their stoichiometry from the known protein-protein interactions in the divisome core. In addition, they updated the “make before break” models of Koch (1984) [52] and Höltje (1998) [47].

The above two approaches underpin the notion that groups of divisome proteins are distributed around the circumference of the cell and that their interactions can be studied. We now know that the modern subassembly is a highly dynamic structure (see below) (Du and Lutkenhaus, 2019 [155]).

### 22.3. Treadmilling of FtsZ Protofilaments

A very remarkable finding is the fact that individual FtsZ filaments are able to treadmill (Loose and Mitchison, 2014 [156]), a phenomenon already known for many years regarding eukaryotic microtubules (remember that the hollow microtubules in a mammalian cell are composed of 13 tubulin filaments). FtsZ treadmilling appeared to be accompanied by peptidoglycan synthesis, thus effecting division (Bisson Filho et al., 2017 [157]). Thus, the subassembly travels along the circumference of the cytoplasmic side of the cell membrane “in both directions with a velocity of about 30 nm/sec”. The lifetime of a subassembly is “about 15 s before it disassembles and new clusters appear” (review and references in Du and Lutkenhaus, 2019 [155]).

**Figure 32:**
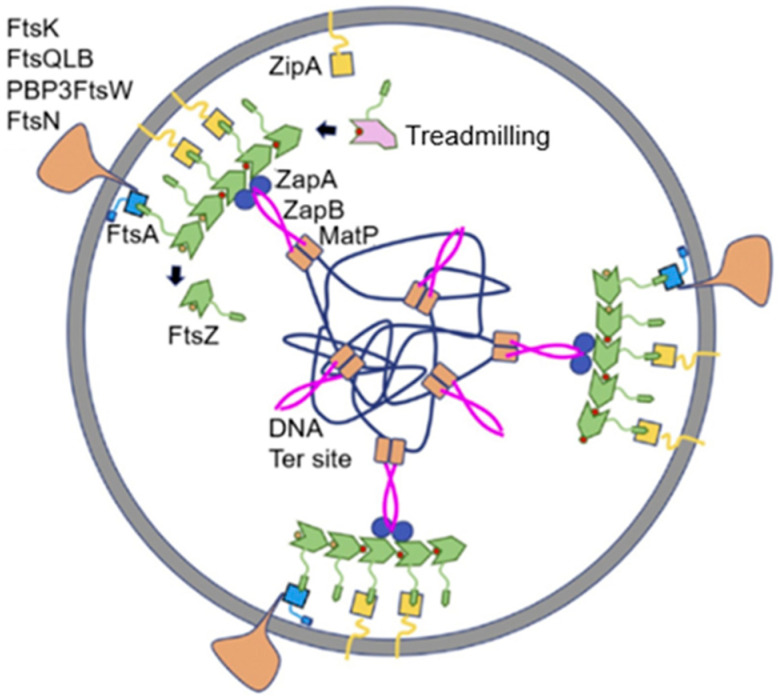
Treadmilling of FtsZ filaments during peptidoglycan synthesis in the direct environment of the terminus of DNA replication. Subassemblies at the cell membrane connected to the peptidoglycan synthetic machinery in the periplasm including FtsK, Fts QLB, FtsWI (PBP3), and FtsN. Internally connected to the terminus of DNA replication (Ter-signal) MatP from FtsZ to MatP via ZapA and ZapB. FtsZ filaments attached to the cell membrane via FtsA and ZipA. The FtsZ filaments are involved in treadmilling in both directions. Source: Modified Figure 4 of [155]. Open access.

**Figure 33:**
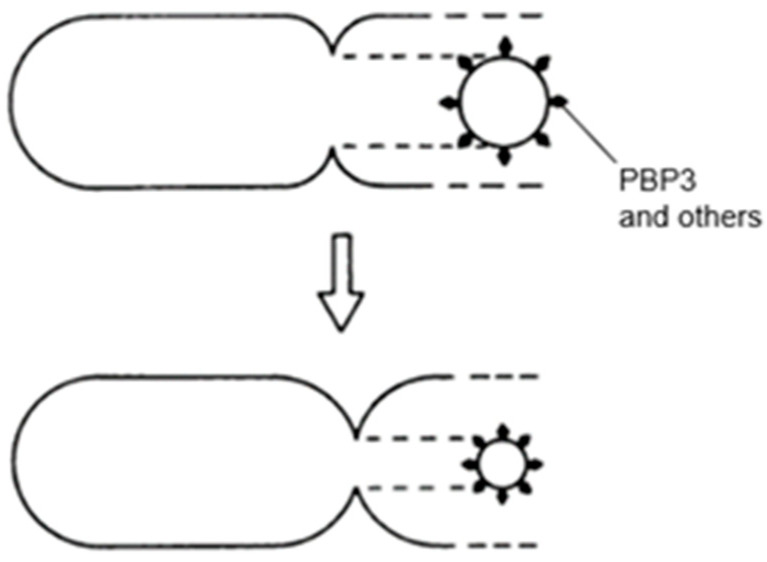
Closer packing of PBP3 and possible other enzymes involved in constriction (♦). As argued in [57], the number of lozenges remains constant. Source: Modified Figure 7 of [57]. Copyright © American Society for Microbiology.

Treadmilling subassemblies are depicted in Figure 32. Here, peptidoglycan synthesis is carried out by the couple PBP3 (transpeptidase) and FtsW (transglycosylase). FtsK and FtsQLB are involved in recruitment, whereas FtsQLB and FtsN play a role in regulation. The FtsZ protofilament has a very flexible binding to the cell membrane via ZipA, whereas FtsA connects FtsZ to the divisome core (review: Du and Lutkenhaus, 2019 [155]). I may recall a model we made in 1989 (Figure 33; Wientjes and Nanninga, 1989 [57]) and I will quote: “Successive stages in constriction. Since the peptidoglycan-synthesizing activity in the leading is constant during constriction, the concentration of enzymes in the leading edge will increase as the constriction process continues. This is depicted in this drawing as a closer packing of the symbol ♦, which represents PBP3 and possible other enzymes involved in constriction”.

A completely different higher-order organization of the divisome has been proposed by Söderström and co-workers (Söderström et al., 2017 [158]). In their model, the divisome is composed of three concentric rings; the central one spans the cytoplasm and cell membrane and encompasses FtsZ, FtsA, and ZipA, thus representing PPS and PIPS. This ring is sandwiched between a ring composed of core proteins without cytoplasmic partners and a cytoplasmic ring contacting the chromosome.

Since the subassemblies encompass a number of cellular compartments, the required interactions are presumably manyfold. At the moment, it seems too complicated to attempt an answer, unless I have missed something (which is not unlikely).

## 23. On the Origin of Confocal Scanning Light Microscopy in Amsterdam

### 23.1. Structure of the Nucleoid

In the beginning of this article, I referred to freeze-fracture experiments with *B. subtilis* with the aim to assess the reliability of chemical fixation (Nanninga, 1969 [20]). This was followed by a focus on mesosomes, with the final conclusion that they arise by chemical fixation (Nanninga, 1971 [15]). In 1969, I was also interested in the appearance of fracture faces of cell membranes after employing prior chemical fixation (RK-fixation; see above). It was shown that cell membranes fractured normally and that their morphology was not visibly altered. However, the nucleoid could not be discerned convincingly (no chemical fixation) unless cells were first fixed with osmium tetroxide. Of course, it could not be concluded that DNA is not there (like mesosomes). Perhaps not surprisingly, the nucleoid’s morphology depended strikingly on the chemical fixation method used (Woldringh and Nanninga, 1976 [159]). Though we admired the phase-contrast light microscopic images of Mason and Powelson (1956) [160], the optical resolution was too low to help us with the interpretation of electron microscopic images. So, we felt stuck.

### 23.2. Origin of Confocal Scanning Laser Microscopy in Amsterdam

Then I came upon a paper of Lemons and Quate (1975) [161], which was about scanning acoustic microscopy and its applications to medical biology. Would this be a method to bridge the gap between light and electron microscopy? I proposed to Fred Brakenhoff to visit them and to have look at the instrument. Why Fred (G.F.) Brakenhoff?

This has to do with the structure of the department in Amsterdam. When at the Technical University of Delft, Woutera van Iterson was accustomed to work with physicists who were involved in designing electron microscopes. With this in mind, I introduced to her a physicist who had just finished his Ph.D. studies, the idea also being not to be fully dependent on a complicated commercial scientific apparatus for our research. So, he became a member of the staff.

**Figure 34:**
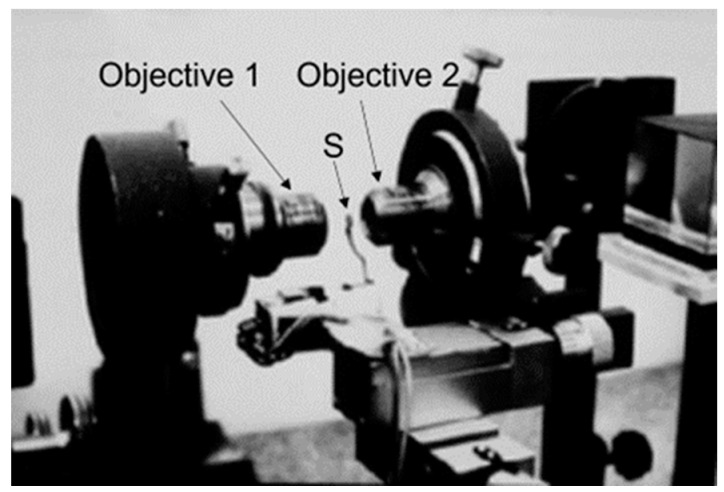
First prototype of a confocal microscope in Amsterdam. Around 1976. The specimen (S) is present in the common focus of objective 1 and objective 2; hence the term confocal.

Brakenhoff went to Lemons and Quate at the Stanford University, California, USA and worked with the acoustic confocal microscope. Thereafter, he came to a remarkable conclusion: it would be better to make an optical equivalent. The instrument was constructed in our department (Brakenhoff et al., 1979 [162]). It had a scanning table instead of a scanning beam in modern instruments. Initially, the operation of the scanning table was conducted by a spare part of a record player, and the whole instrument rested on an inflated car tyre (these are memories of a biologist). The first prototype is depicted in Figure 34, and a later version can be found in [162].

Sheppard and Choudhury (1977) [163] calculated that the theoretical gain in optical resolution would be $\sqrt{7}$ and I considered this a significant improvement, but was it useful for our purpose?

**Figure 35:**
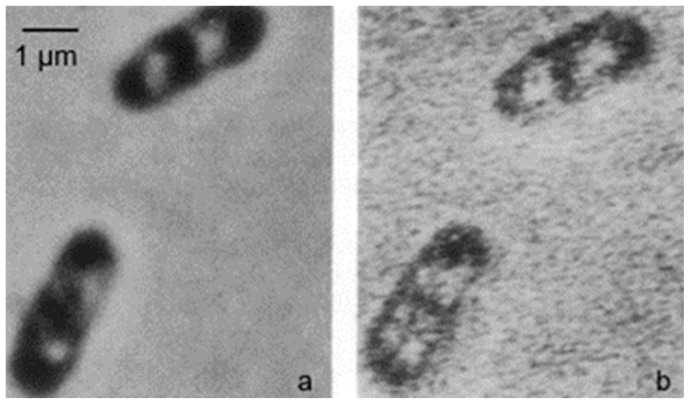
Phase-contrast image (**a**) and confocal transmission image (**b**) of *E. coli* H266. Doubling time 21 min. Bar, 1 µm. Source: Modified Figure 3a,b [164]. Copyright © American Society for Microbiology.

**Figure 36:**
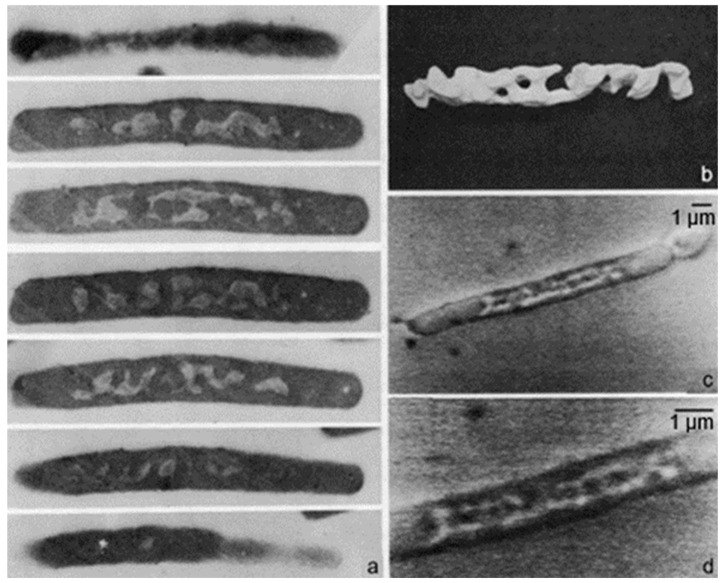
Comparison of electron microscopic thin sections (RK-fixation) (**a**) with a nucleoid reconstruction (**b**) and CSLM transmission image of a living cell of *E. coli* LE316 *gyrB* shifted from 30 to 42 °C and grown for 60 min at 42 °C (**c**,**d**). The ladder-like nucleoid resembles the model in (**b**). Bar, 1 µm. Source: Modified Figure 2 of [164]. Copyright © American Society for Microbiology.

In attempting to answer this question, we made an exhaustive study by comparing phase-contrast light microscopy, confocal scanning light microscopy, and electron microscopy of living and chemically fixed *E. coli* cells (Valkenburg et al., 1985 [164]). I will not go into details but show that a higher resolution indeed was obtained. I will give two examples of the above paper. In Figure 35, a phase-contrast image can be compared with a CSLM image of *E. coli* B/r H266 (doubling time 21 min). The latter implies multifork replication, revealing a complex image of the nucleoid, notably in the CSLM cells. The next group of images represent *E. coli* LE316 *gyrB* grown at the non-permissive temperature of 42 °C. Filaments are formed and the nucleoids are not segregated. Compared are chemically fixed cells in Figure 36a and living cells in Figure 36c,d. Of the serial sections, a model was made by cutting the contours (paper) of the nucleoids and they were piled and subsequently photographed (Figure 36b). The latter model (b) of the nucleoids at the non-permissive temperature strikingly resembles those in living cells (Figure 36c,d). So, the gain in resolution, we felt, was demonstrated. Note that the confocal images are based on transmission microscopy and a contrast obtained by light absorption (as contrasted to fluorescence microscopy).

How to continue from there on? In fact, this was dictated by the emergence of fluorescent probes and lasers and the development of computer technology, three factors that did not yet exist so dominantly when we started with confocal scanning light microscopy in the seventies of the previous century. We therefore altered our approach.

### 23.3. Three-Dimensional Confocal Scanning Laser Microscopy

**Figure 37:**
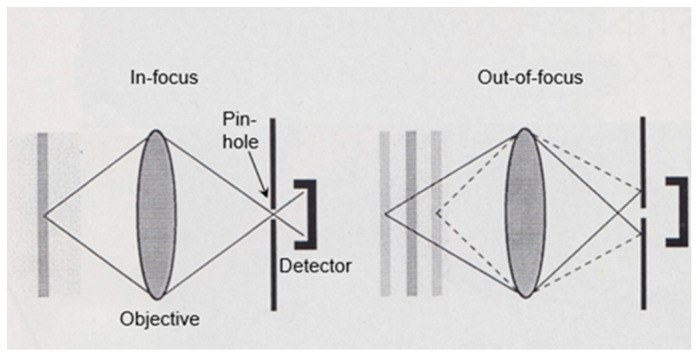
Effect of a pinhole on sharpness of focus. Left, in-focus layer and right, out-of-focus layers that do not contribute to the image. Digital layers arise by scanning, and they can be piled to allow, for instance, a stereo effect.

The above new possibilities led us to design a computer-controlled confocal microscope (van der Voort et al., 1985 [165]). Essential is the use of pinholes, that is, an illuminated pinhole is focused on to the specimen. Next, the light coming from the specimen is focused on a second pinhole above a photodetector which digitizes the light signal. The importance of a pinhole is illustrated in Figure 37; it shows how the microscope is focused on a thin digital slice by the detection pinhole, thus eliminating out-of-focus information. The mechanical scanning device controlled by a microprocessor produces a series of optical sections (slices). In this way, stacks of images are stored in the computer, and they are thus available for all kinds of image-processing techniques. Screening the stacks from different angles can produce stereo images (Brakenhoff et al., 1985 [166]).

**Figure 38:**
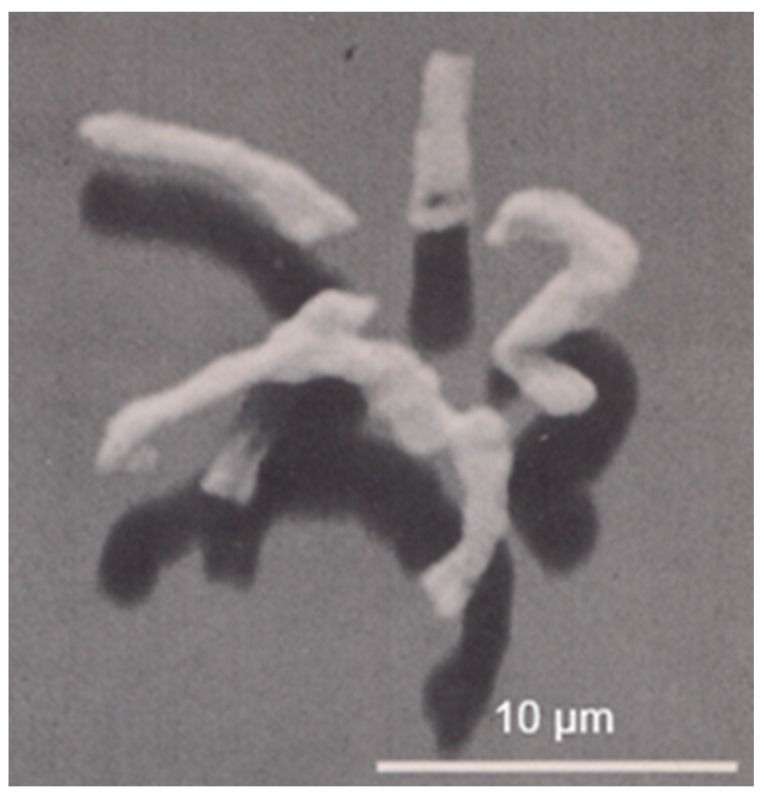
Metaphase chromosomes (2n = 6) with a radial arrangement from a root-tip cell of *Crepis capillaris*. Various filtering procedures, a ray-tracing technique and artificial shadowing were applied to a 3-D reconstruction. Source: Figure 2a of [168]. © Company of Biologists Ltd. [168].

However, showing such images to an audience appeared very cumbersome. Hence, Hans van der Voort made “clean” solid models of fluorescent objects and added a virtual light source to the whole. The latter created shadows to provide for an impression of depth (van der Voort et al., 1989 [167]). An example is shown in Figure 38 (Oud et al., 1989 [168]). Depicted are *Crepis capillaris* (smooth hawksbead) metaphase chromosomes (2n = 6). We asked the question whether chromosomes have a fixed position towards each other in mitotic anaphase. The answer can be found in the above paper. We embarked on a fruitful project on plant chromosomes with the expert insights of Oof (J. L.) Oud. Also, further optical and image processing techniques, as well as the design of unique fluorescent probes, have been developed. Notably, by my very able successor T. W. J. (Dorus) Gadella, who now occupies the chair of Molecular Cytology at the University of Amsterdam. Unfortunately, this is beyond the scope of this paper.

## 24. Epilogue

Writing this paper has been a journey, and what has been written down is, of course, subjective. I was asked to present my personal recollections, so I can perhaps be forgiven for my subjectivity.

**Figure 39:**
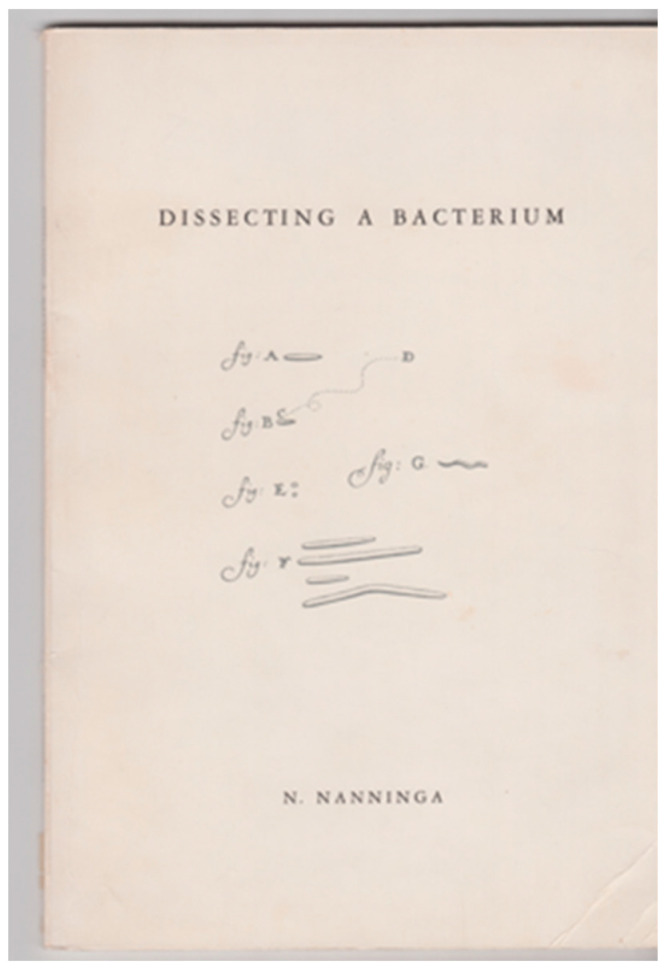
Cover of the Ph.D. thesis of N. Nanninga on 6 May 1970. University of Amsterdam.

The title of my contribution contains the phrase “little animals”, which derives from van Leeuwenhoek (1683). They were little animals because they could move, unlike plants. Actually, the first prokaryote described by van Leeuwenhoek was probably a cyanobacterium. This I have discussed in the blog Small Things Considered: “Van Leeuwenhoek’s freshwater microorganisms in 1674. *Spirogyra* or *Anabaena*”? I take the liberty to add as a last figure the cover of my thesis in 1970, which started with the dissecting of the little animals (Figure 39).

## Figures and Tables

**Table 1:** Muropeptide composition of an exponentially growing *E. coli* MC4100 culture.

Muropeptide	Fraction of Incorporated [^3^H]-Dap
Tri	0.4(0.1)
Tet	54.4(0.9)
Pen	1.0(0.1)
Tri-Lys	3.1(0.1)
Tet-Tri-DAP	0.4(0.1)
Tet-Tri	2.4(0.1)
Tet-Tet	28.8(0.4)
Tet-Anh	1.0(0.3)
Tet-Pen	2.0(0.0)
Tet-Tri-DAP-Lys	0.3(0.0)
Tet-Tet-Tri	0.4(0.0)
Tet-Tri-Lys	0.8(0.2)
Tet-Tet-Tet	1.6(0.1)
Tet-Tri-Anh	0.4(0.1)
Tet-Tet-Tet-Tet	0.1(0.0)
Tet-Tet-Tri-Lys	0.3(0.2)
Tet-Tet-Anh	1.2(0.1)
Tet-Tet-Tri-Anh	0.2(0.1)
Tet-Tet-Tet-Anh	0.4(0.1)
Dap-Dap	Degree of cross-linking 0.4(0.0)
	Disaccharide peptides (% of total) containing lipoprotein 4.1(0.3)

## Data Availability

Not applicable.
